# A Novel, Portable and Fast Moisture Content Measuring Method for Grains Based on an Ultra-Wideband (UWB) Radar Module and the Mode Matching Method

**DOI:** 10.3390/s19194224

**Published:** 2019-09-28

**Authors:** Chi Zhang, Zhichao Shi, Haiying Yang, Xiaoguang Zhou, Zidan Wu, Digvir S. Jayas

**Affiliations:** 1 Department of Automation, Beijing University of Posts and Telecommunications, Beijing 100876, China; zhangchi87@bupt.edu.cn (C.Z.); shizhichao@bupt.edu.cn (Z.S.); yang_haiying@bupt.edu.cn (H.Y.); 2 Department of Biological and Agricultural Engineering, Jilin University, Changchun 130012, China; 3 Department of Biosystems Engineering, University of Manitoba, Winnipeg, MB R3T 2N2, Canada; digvir.jayas@umanitoba.ca

**Keywords:** moisture measurement, grain, ultra-wideband radar, sensors, mode matching method

## Abstract

To perform fast and portable grain moisture measurements under field conditions, a novel moisture sensor was designed, which consisted of a coaxial waveguide, a circular waveguide, and an isolation layer. The electromagnetic characteristics of the sensor were simulated and measured. The analytical model, which represented the relationship between the reflection coefficient of the sensor and the complex permittivity of grain, was established by using the mode matching method. The reflection coefficient of the sensor was measured by using an ultra-wideband (UWB) radar module, and the moisture content of grains was calculated from the complex permittivity by using density-independent model. To verify the performance of the proposed method, wheat, rough rice, and barley were taken as examples. The measured results in the range from 1.0% to 26.0%, wet basis, agreed well with the reference values (R^2^ was more than 0.99), and the maximum absolute errors for wheat, rough rice, and barley were 1.1%, 1.0%, and 1.4%, respectively. In addition, the effect of isolation layer was discussed. Both the simulation results and the experimental results showed that the isolation layer improved the stability of sensor.

## 1. Introduction

Accurate determination of moisture content of grains is of great importance in grain trading, transportation, storage, and processing. Especially during storage, grain moisture plays an important role in the safe storage of grains [1,2]. Using the convection oven to dry the grain and measuring the loss of mass to determine the grain moisture is the standard method of grain moisture measurement in several countries because of its high precision and high reliability [3,4,5], however, it has obvious disadvantages of being time-consuming and cumbersome. In order to achieve fast and precise moisture determination, many studies have been conducted to determine the moisture of grains by using near-infrared reflection methods [6,7,8]. However, the penetration depth of infrared is shallow, and the measured result is easily affected by ambient light and impurities attached to the grain particles, therefore, the method is most often applied to grain moving on a conveyor belt in a processing plant, where the layer of grain is thin and the environment is relatively clean [9].

With good penetrability, the microwave has been widely studied for measuring the moisture of grain [10,11,12]. Microwave method indirectly calculates the moisture content of grain by measuring the complex permittivity, which is related to grain moisture. The complex permittivity is intrinsic properties of grain, and it is usually expressed as $\varepsilon =\varepsilon^{\prime }-j\varepsilon^{\prime \prime }$. The real part $\varepsilon^{\prime }$ is called dielectric constant, and the negative number of the imaginary part $\varepsilon^{\prime \prime }$ is called the loss factor. The complex permittivity of grain is the equivalent complex permittivity of a mixture of grain kernels and air. When the grain kernels’ size is much smaller than the wavelength, the mixture is an effective medium, which means grain can be regarded as a homogeneous medium. The size parameter can be used to evaluate the size of grains relating to the wavelength, which is defined as 2πr/λ, where r is the radius of particles and λ is the wavelength [13]. Taking wheat as an example, the maximum radius of the grain kernels is less than 4 mm [14]. For electromagnetic waves with a frequency less than 4.8 GHz, the wavelength is greater than 62.5 mm. Consequently, the size parameters of grains were less than 0.4. It means that the grain kernels are small enough relative to the wavelength to gain as a homogeneous medium described by one complex permittivity. Besides, the influence of bulk density on measured results was a main problem faced by microwave grain moisture measuring technology. Fortunately, the dielectric constant and loss factor of grain were both related to the moisture content and the bulk density. Consequently, two equations can be established. One describes the relationship among the dielectric constant, the moisture content, and the bulk density. The other one describes the relationship among the loss factor, the moisture content, and the bulk density. Combining the two equations to eliminate the bulk density term, an equation can be obtained for calculating grain moisture from the dielectric constant and the loss factor. Based on the idea and many experimental data, Trabelsi et al. [15,16,17] have proposed a density-independent calibration function as Equation (1) and a temperature compensation function as Equation (2):

(1) $$\psi =\sqrt{\frac{\varepsilon^{\prime \prime }}{\varepsilon^{\prime }\left(a_{f}\varepsilon^{\prime }-\varepsilon^{\prime \prime }\right)}}$$

(2) $$\psi =a_{1}T+a_{2}W+a_{3}$$

where ψ is a density-independent calibration function, which is independent of the bulk density and is linearly related to the moisture content and temperature. At any given frequency, the value of ψ depends only on the grain complex permittivity. $a_{f}$ is a frequency factor, which is related to the microwave frequency. It is a constant for a given grain sample at a given frequency. In the literature [15], the linear relationship between expressions $\varepsilon^{\prime }/\rho$ and $\varepsilon^{\prime \prime }/\rho$ was found. ρ is the bulk density. The parameter $a_{f}$ was obtained from a complex plane consisting of $\varepsilon^{\prime }/\rho$ and $\varepsilon^{\prime \prime }/\rho$, and the parameter $a_{f}$ equaled the slope of the linear regression function of $\varepsilon^{\prime }/\rho$ and $\varepsilon^{\prime \prime }/\rho$. T is temperature (°C), W is the percentage of moisture (wet basis). $a_{1}$, $a_{2}$ and $a_{3}$ are three undetermined coefficients. The model has been proven effective for the moisture determination of wheat, corn, soybeans, and peanuts (with a standard error of less than 0.97%) [1]. In particular, a shelled peanut moisture determination device was developed by using free-space method (the standard error less than 0.55%), and successfully applied it in the peanut drying field [18,19,20,21]. The free space method can be used to calculate the complex permittivity of grain by measuring the attenuation and phase change of the microwave propagated through the grain. In this condition, only the microwave which transmits through the grain was related to the permittivity of grain. If the size of transverse of the sampler holder is not large enough, there will be great diffraction at the edges of the sample holder. A large part of the microwave energy will pass through the edges of the sample holder instead of penetrating it. To prevent the diffraction at the edges of the sample holder and the multiple reflections in the sample holder, the size of the cross-section and the thickness of the sampler holder must be large enough. The sizes of the sample holder in their studies [20,21] were 22.2 cm × 13.3 cm × 21.9 cm (4.5 L) and 4.4 cm × 4.4 cm × 12 cm (243 mL), respectively. This makes the measuring device bulky and difficult to carry, limiting its scope of application.

The open-ended waveguide technique is a type of microwave reflection method. Due to the different impedances between the sample and the waveguide, the discontinuous impedance at the interface between the waveguide and the measured object will cause microwave reflection. The intensity and phase of the reflected microwaves are related to the impedance of the sample, and the impedance of the sample is related to its complex permittivity. As a result, the measurement of the complex permittivity of the sample can be achieved by measuring the reflection coefficient of the microwaves at the interface between the waveguide and the sample. During the measuring process, no diffraction phenomenon exists, and only a small volume of sample can achieve the measurement. Consequently, compared to the free space method, the open-ended waveguide reflection method is very suitable for miniaturizing the sensor. The open-ended coaxial waveguide [22], the open-ended circular waveguide [23], the open-ended rectangular waveguide [24], and the open-ended substrate integrated waveguide (SIW) [25] are usually adopted. The SIW can be regarded as a rectangular waveguide, which has the advantage of small size. Compared to the open-ended circular waveguides, the open-ended rectangular waveguides, and the open-ended SIW, the open-ended coaxial waveguides have two outstanding advantages. First, the open-ended coaxial waveguide has no cut-off frequency, so it is especially suitable for wideband measurements. Secondly, the structure of the open-ended coaxial waveguide is the same as the coaxial cable. When the waveguide connects the coaxial cable, the connector is easy to be designed and manufactured. Using the circular waveguide or the rectangular waveguide needs to design the impedance matching structure and the electromagnetic field excitation structure for connecting the coaxial cable. Currently, the open-ended coaxial waveguide is widely used in liquid broadband complex permittivity measurement [26,27,28].

**Figure 1:**
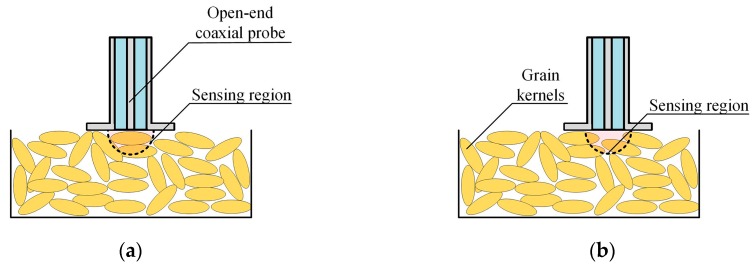
Schematic of the sensing region of an open-ended coaxial probe measuring grain samples: (**a**), the center of the probe connecting to a grain kernel; (**b**), the center of the probe connecting to pore among grain kernels.

However, there are many problems in applying the open-ended coaxial waveguide directly to the grain moisture measurement field. Firstly, the existing open-ended coaxial waveguide can only be applied to small-sized particles. The size of the grain kernel is too large for the small sensing region of the open-ended coaxial probe. The complex permittivity of grain is the equivalent complex permittivity of a mixture of grain kernels and air. If the volume ratio of grain to air in the sensing region is different from the actual ratio, the measurement will have a large error. As shown in Figure 1a, when the volume ratio of grain to air in the sensing region is larger than the actual ratio, the measured complex permittivity will be larger than its actual value. As shown in Figure 1b, when the volume ratio of grain to air in the sensing region is smaller than the actual ratio, the measured complex permittivity will be smaller than its actual value. Since the volume ratio of grain to air in the sensing area is random, the measured complex permittivity is significantly unstable. This makes the method unusable for grain. Therefore, small size of the sensing region is the primary problem in applying the open-ended coaxial probe to the field of grain moisture measurement. The sensing region increases as the radius of the outer conductor increases [29], so the sensing region can be expanded by adjusting the dimensions of the open-ended coaxial probe. In addition, another waveguide which connects with the coaxial waveguide can be designed to modify the impedance of the microwave in the grain. With proper dimensions, there will be more microwave energy entering the grain from the coaxial waveguide. Consequently, the depth of the sensing region can become larger. In this paper, by expanding the outer conductor radius of the coaxial waveguide and loading the grain sample in a circular waveguide, the expansion of the sensing region was realized. As a result, the improved open-ended coaxial probe can be suitable for the field of grain moisture measurement. Secondly, the environment (such as the ground, container) easily affects the measurement. Fortunately, the grain sample was loaded in a circular waveguide in this paper, and the metal boundary can prevent the measurement from the interference of the environment. Thirdly, the traditional coaxial probe models are not suitable. There are capacitive, quasi-static, and Taylor-series models to describe the relationship between the complex permittivity and the admittance of the coaxial end face [30]. However, these models are proposed under two hypotheses. One is an infinitely large grain sample is filled in a half-space to avoid reflection signals affecting the measurement. This is impractical for the grains which have small dielectric constants and low loss factors. The other is that only one microwave mode—transverse electromagnetic mode (TEM)—exists in the open-ended coaxial probe. Although the open-ended coaxial probe is designed to fit single-mode transmission conditions, the high-order mode will be generated due to the discontinuity of impedance at the end face of the coaxial waveguide. These high-order modes have a great influence on the measurements. To solve this problem, the mode matching method [31], an effective approach for analyzing the characteristics of microwaves in discontinuous waveguides, was adopted to build the model.

In this paper, a novel, portable, and fast grain moisture content measuring method is proposed, which overcomes the problems of the open-ended coaxial waveguide application in the grain moisture measurement field. Firstly, a grain moisture sensor was designed by combining the coaxial waveguide with a circular waveguide. The circular waveguide can be used as a sampler or the sample holder, and it can prevent the measurement from the environmental interference. Secondly, based on the mode matching method [31], a new model was developed, which could precisely describe the relationship between the complex permittivity of grain and the reflection coefficient of the sensor. As the grain moisture increased, the real part and the absolute value of the imaginary part of the complex permittivity increased, and the magnitude of the corresponding reflection coefficient decreased and the phase decreased. Thirdly, a broadband reflection coefficient measuring method by using the ultra-wideband (UWB) radar module was proposed. Finally, taking wheat, rough rice, and barley as examples, the accuracy and stability of the method were verified. In addition, the paper discussed the influence of the pressure of the sensor on the accuracy of the proposed method.

## 2. Method

### 2.1. Design of the Grain Moisture Sensor

**Figure 2:**
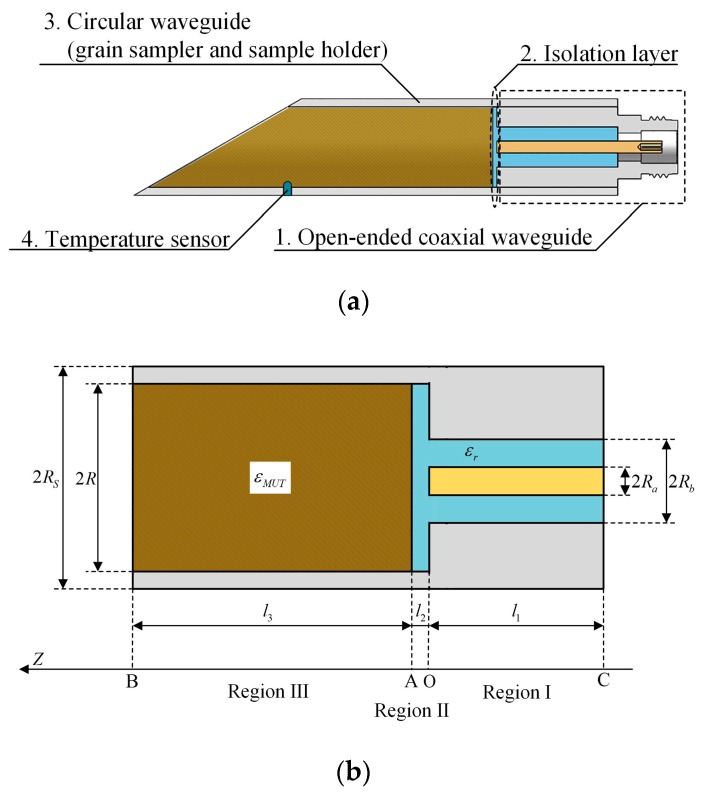
Schematic of the proposed grain moisture sensor: (**a**), schematic of structures; (**b**), sketch of dimensions.

Due to the wide bandwidth, no diffraction and simple structure of the open-ended coaxial waveguide, a novel grain moisture sensor was designed. The sensor combined a circular waveguide (grain sampler and sample holder) with the open-ended coaxial waveguide in order to make the sensor suitable for grain moisture measurement in field condition. The schematic of the sensor is shown in Figure 2 and the dimensions are shown in Table 1. The sensor consisted of an open-ended coaxial waveguide, a circular waveguide, an isolation layer, a temperature sensor, and the type-N microwave connector. The sensor’s main features were as follows:

*The open-ended coaxial waveguide*. The type-N microwave connector was directly connected to the open-ended coaxial waveguide. The inner conductor was made of gold-plated brass, the outer conductor was made of 304 stainless steel, and the gap between inner and outer conductors was filled with Teflon. Since the dielectric constant of Teflon is approximately a constant ($\varepsilon_{r}=2.1$) over wideband [32], using Teflon to fill the gap can easily make the impedance of the coaxial waveguide be a constant over a wide range of frequency. The dimensions were chosen to make the characteristic impedance equal to 50 Ω and only TEM mode can transmit in the frequency range of 0-5 GHz.

*The isolation layer*. The end face of the coaxial waveguide was covered with an isolation layer, which was made of Teflon. The isolation layer had two functions. First, it can protect the plating of the inner conductor. The conductivity changes can be avoided in long-term use thus improving the stability of the sensor. Second, it can improve the accuracy. Without the isolation layer, a conductive path will be formed between the inner and outer conductors through grain kernels. Unfortunately, the characteristic of the path is uncertain, because of the random distribution of grain kernels. As a result, without the isolation layer, there will be a large standard deviation in the measurement of the same sample.

*The circular waveguide (grain sampler and sample holder)*. The circular waveguide was made of 304 stainless steel tubing, which was connected to the open-ended coaxial waveguide. The front of the circular waveguide was cut to 30 degrees, which made it easy to insert into the grain. The inner diameter was designed to make all microwave modes attenuate in the frequency range of 0-5 GHz. This allowed the electromagnetic field to exist in a limited region. Consequently, the measurement needs a small amount of grain sample, and the environment outside does not affect the test results. This can improve accuracy. The volume of the sampler was about 30 mL. Consequently, our proposed method only needs 30 mL grain to complete the measurement. Besides, the circular waveguide can be used as a sampler and the sample holder, since the inside of the circular waveguide was empty. This can make the sensor more convenient to use. Instead of sampling the grain and loading the grain sample into the sample holder, the proposed sensor can be inserted into the grain bulk, the measuring processes of sampling grain and loading the sample into the sample holder can be accomplished simultaneously. Therefore, the proposed sensor can simplify the measurement process and increasing the efficiency.

*Temperature sensor*. The PT100 platinum resistance thermometer (diameter: 2 mm, height: 5 mm) was placed far away from the isolation layer, which made the coaxial waveguide not be affected by the shell of the temperature sensor. In addition, the temperature sensor was isolated from the sampler by a 2 mm thick ABS plastic, which avoided the influence of the sampler’s temperature on measuring grain’s temperature.

### 2.2. Simulation and Measurement of the Proposed Sensor

**Figure 3:**
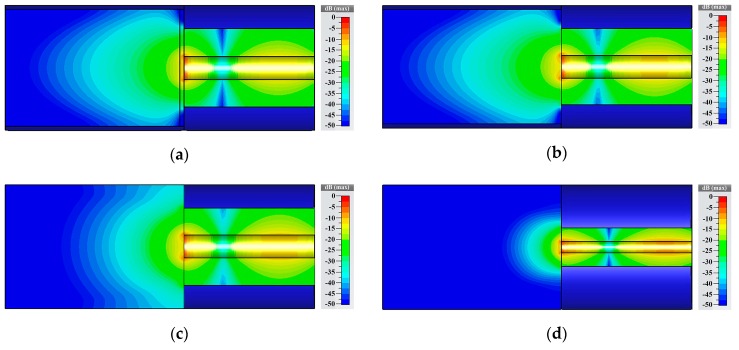
Electric field simulation results at 4 GHz: (**a**), the proposed sensor with isolation layer; (**b**), sensor without isolation layer; (**c**), coaxial sensor; (**d**), coaxial sensor with small radii of inner and outer conductor.

Taking $\varepsilon_{MUT}=2.563-j0.318$ as the complex permittivity of the measured object, the electromagnetic field of the proposed sensor was simulated by CST (Dassault Systemes, Stuttgart, Germany). The complex permittivity was derived from the complex permittivity of white hard winter wheat at 4 GHz with a moisture content of 12.6%, which was adopted as a grain sample in this paper. Figure 3 showed the electric field at 4 GHz. Figure 3c,d shows conventional open-ended coaxial probes. The inner and outer conductor radii were 3 mm and 10 mm in Figure 3c, respectively. The inner and outer conductor radii were 1.5 mm and 5 mm in Figure 3d, respectively. The region where the electric field was greater than −30 dB was the sensor’s sensing region. The interference at the electric field above −30 dB can be detected by the UWB radar module with a dynamic range of 60 dB adopted in this paper. The depth of sensing region of Figure 3c was 18 mm, and the depth of sensing region of Figure 3d was 9 mm. Therefore, the sensing region of the open-ended coaxial probe can be increased by increasing the radii of the inner and outer conductors. However, there is a limit to the inner and outer conductor radii. Their radii are limited by the single mode transmission condition as shown in the following [33]:

(3) $$R_{a}+R_{b}<\frac{C_{0}}{\pi \sqrt{\varepsilon_{r}}f_{\max}}$$

where $C_{0}$ is the velocity of light, and $f_{\max}$ is the maximum frequency of sensor adopted. This indicates that the greater is the sum of the inner and outer conductor radii, the lower is the maximum frequency of the sensor used. In order to maximize the sensing region, we chose the maximum of the sum of inner and outer conductor radii, which met the single mode transmission condition in the frequency range of 0–5 GHz. Comparing to Figure 3c, Figure 3b added a circular waveguide with an inner radius of 15 mm connected to the coaxial waveguide. The circular waveguide could be seen as the sample holder. The wave impedance of the grain inside the circular waveguide was different from the wave impedance of the grain in free space. Choosing proper dimensions of the circular waveguide can change the wave impedance of the grain sample, which made more energy of electromagnetic wave enter into the grain sample. As a result, the sensing region of the sensor can be enlarged. In Figure 3b, the depth of the sensing region was 26 mm, which was 13 mm more than the open-ended coaxial probe shown in Figure 3c. In addition, since the grain sample was in free space in Figure 3c, a part of the energy of electromagnetic wave leaked to the upper and lower space. If the environments interacted with this part of the energy, it would introduce environmental interference. In contrast, the electromagnetic wave in Figure 3b was enclosed in the circular waveguide. Consequently, external interference was avoided. Figure 3a was the proposed sensor in this paper. Comparing to Figure 3b, the proposed sensor was added the isolation layer. The main function of the isolation layer was to prevent the coaxial inner conductor from direct contact with the grain kernels. This can protect the inner conductor’s plating. In addition, it can reduce the standard deviation of the measurement and improve the stability (the details were analyzed in the Section 5.5). The depth of the sensing region of the proposed sensor was 25 mm, which was only 1 mm less than sensor without isolation layer. It indicated that the isolation layer hardly influenced the depth of the sensing region. This was mainly because Teflon had a small loss factor and its dielectric constant was close to grain. As a result, the isolation layer improved the stability and did not significantly affect the electromagnetic wave propagating into the grain sample.

The temperature sensor used in this paper was fixed on the edge of the opening of the circular waveguide. The electric field was less than −50 dB. The electric field intensity at the position of the temperature sensor was very small. In addition, the temperature sensor had a metal shell, which can produce electromagnetic shielding. Consequently, the electromagnetic field in the proposed sensor did not interfere with the temperature sensor.

To compare simulation and measurement of the proposed sensor’s S-parameter, the decanol was adopted as standard material. Because the complex permittivity of decanol was very similar to grain and it can be measured by a 85070E dielectric probe (Keysight Technologies, Santa Rosa, CA, USA). Consequently, the decanol’s complex permittivity measured by the 85070E was brought into the simulation. The S-parameter of the sensor was measured by an E5071C vector network analyzer (VNA) (Keysight Technologies).

**Figure 4:**
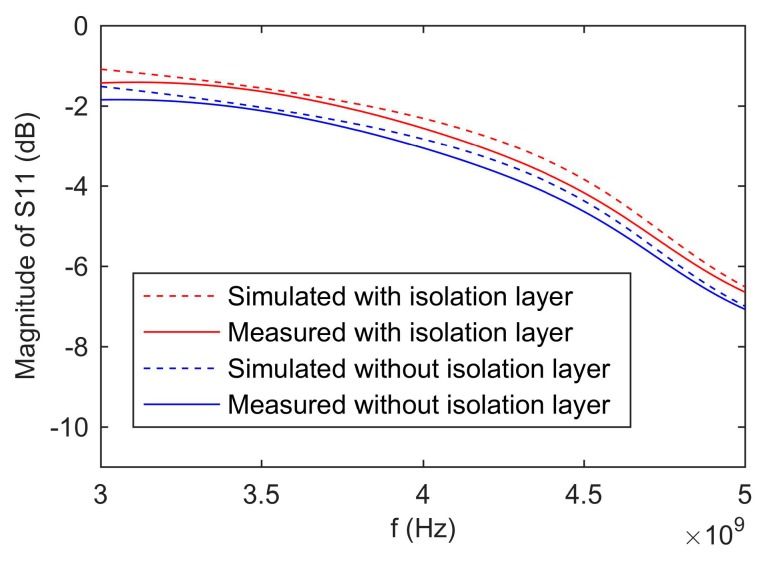
Simulated and measured results of S-parameter of the proposed sensor with isolation layer and without isolation layer.

Figure 4 shows the simulated and measured values of S-parameter of the proposed sensor with isolation layer versus without isolation layer. The measured results were consistent with the simulation results. The differences between the measured and simulation results were caused by the error of the fabrication. Besides, the magnitude of S11 of the sensor with the isolation layer was larger than the sensor without the isolation layer. This was because the isolation layer reduced a part of the electromagnetic wave energy propagating into the sample holder. Consequently, more electromagnetic wave energy was reflected.

### 2.3. Modeling the Sensor Based on the Mode Matching Method

The mode matching method is an effective approach for analyzing the characteristics of microwaves in discontinuous waveguides [31]. At both sides of the discontinuous interface, the transverse electric and magnetic fields can be expanded into combinations of transverse electromagnetic (TEM), transverse magnetic (TM) and transverse electric (TE) modes. The scattering coefficient at the discontinuous interface can be obtained by using boundary conditions and the orthogonality between different modes. In this paper, the electromagnetic field in the coaxial region was expanded into a combination of one TEM mode and *N* TM_0n_ modes. The electromagnetic field in the isolation layer was expanded into a combination of *M* TM_0n_ modes. Applying the mode matching method at the interface between the coaxial waveguide and the isolation layer in the circular waveguide, the model of the proposed sensor can be built as follows (details are given in Appendix A):

(4) y=Xη

(5) $$y=\left(\begin{array}{c} \left(Y^{TEM-I}-{\bar{Y}}_{1}^{TM-II}\right)R_{01}\\ \left(Y^{TEM-I}-{\bar{Y}}_{2}^{TM-II}\right)R_{02}\\ \vdots \\ \left(Y^{TEM-I}-{\bar{Y}}_{M}^{TM-II}\right)R_{M} \end{array}\right)$$

(6) $$X=\left(\begin{array}{ccc} \left(Y^{TEM-I}+{\bar{Y}}_{1}^{TM-II}\right)R_{01} & \cdots & \left(Y_{N}^{TM-I}+{\bar{Y}}_{1}^{TM-II}\right)R_{N1}\\ \vdots & \ddots & \vdots \\ \left(Y^{TEM-I}+{\bar{Y}}_{M}^{TM-II}\right)R_{0M} & \cdots & \left(Y_{N}^{TM-I}+{\bar{Y}}_{M}^{TM-II}\right)R_{NM} \end{array}\right).$$

(7) $$\eta =\left(\begin{array}{c} \Gamma^{TEM-I}\\ m_{1}\\ \vdots \\ m_{N} \end{array}\right)$$

where $\Gamma^{TEM-I}$ denotes the reflection coefficient of the TEM mode in the region I, $m_{i}$ denotes the *i*-th TM mode electric field intensity coefficient in the region I. $Y^{TEM-I}$ and $Y_{i}^{TM-I}$ are the admittances of the TEM mode and the *i*-th TM mode in the region I, respectively. $R_{ik}$ indicates the coupling coefficient between the *i*-th mode in region I and the *k*-th mode in region II. ${\bar{Y}}_{i}^{TM-II}$ indicates input admittances of the *i*-th TM mode at position O. Only the term ${\bar{Y}}_{i}^{TM-II}$ in the matrix X and the column vector y are related to the complex permittivity of the grain, and the other terms are constants determined by dimensions of the moisture sensor. The first element of the column vector is the TEM mode reflection coefficient at position O in the region I. As a result, Equation (4) can describe the relationship between the complex permittivity of the grain and the reflection coefficient of the coaxial waveguide (details about the methods for calculating admittances and coupling coefficients are given in Appendix B).

When the complex permittivity of the substance filled in the sampler is known, it is easy to calculate the reflection coefficient by using Equation (4). However, the matrix X may be not a square matrix. In this paper, we solved this problem by the least squares method as follows:

(8) $$\eta ={\left(X^{T}X\right)}^{-1}X^{T}y$$

When the reflection coefficient is known, it is very difficult to calculate the complex permittivity of the grain, because the analytical equation for calculating the complex permittivity from the reflection coefficient cannot be obtained. Fortunately, in 3.1–4.8 GHz range, the ranges of complex permittivity of wheat, barley, and rough rice are bounded ($\varepsilon^{\prime }\in \left[1,6\right]$, $\varepsilon^{\prime \prime }\in \left[0,1\right]$) [1]. For bounded problems that cannot be solved analytically, the look-up table method can quickly give an approximate solution. Low time consumption is the advantage of the method, while large memory consumption is a disadvantage. Its accuracy depends on the step size of the discretization. The smaller the step, the higher the accuracy. Since the range of the complex permittivity is limited, the problem can be solved by the look-up table method. In this paper, the range of complex permittivity was discretized in steps of 0.001, and reflection coefficients corresponding to each complex permittivity was calculated. We created a table containing all complex dielectric constant values and corresponding reflection coefficients. When the reflection coefficient was known, its corresponding complex permittivity could be obtained by looking up the table to find the closest item.

**Figure 5:**
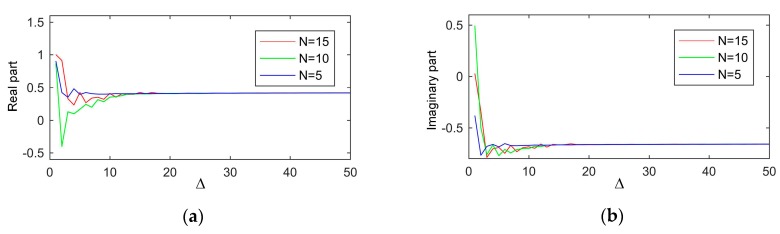
The influence of N and M on the reflection coefficient: (**a**), real part; (**b**), imaginary part.

Determining *N* and *M* in Equation (4) is very significant. If these are infinity, Equation (4) is an accurate model describing the proposed sensor. However, considering the computational complexity and precision, the appropriate values should be chosen. Since the radius of the isolation layer is much larger than the distance between the inner and outer conductors of the coaxial waveguide, the intensity of high-order modes in isolation layer is larger than that in the coaxial waveguide at the same frequency. Consequently, *M* should be greater than *N*. To determine the values of *N* and *M*, we took $\varepsilon_{MUT}=2.563-j0.318$ (the data from white hard winter wheat adopted in this paper) as an example to study the influence of *N* and *M* on the reflection coefficient at 4 GHz. The result is shown in Figure 5. The Δ denotes for *M-N*. First of all, the reflection coefficient became stable when Δ was large enough. Secondly, the larger the value of *N*, the larger the value of Δ was needed. Thirdly, regardless of *N*, the reflection coefficient converged to the same value as Δ increased. Considering the computational complexity and precision, *N* equaled 5 and Δ equaled 20 (*M* equaled 25) in this paper.

### 2.4. The Reflection Coefficient Measuring Method Based on the UWB Radar Module

**Figure 6:**
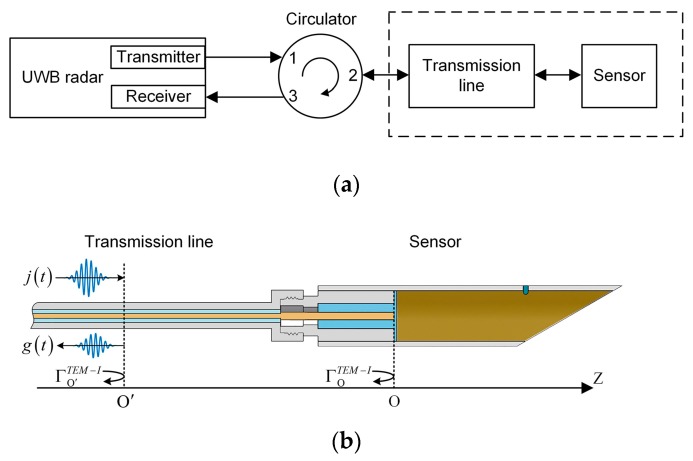
Diagram of reflection coefficient measuring approach: (**a**), block diagram; (**b**), detailed structure in the dashed line box.

To achieve a portable device to measure wideband reflection coefficient, a new approach based on a UWB radar module was proposed in this paper. The diagram is shown in Figure 6a. It consisted of a UWB radar module, a circulator, and a 4 m long transmission line. The radar module (P440, Humatics Inc., Waltham, MA, USA) used in this paper can generate 3.1–4.8 GHz UWB signals, and the sampling interval of the receiver was 61 ps (matching the Nyquist sampling theorem). The circulator (UIYCC2528A, UIY Inc., Shenzhen, China) used in this paper had a 0.5 dB insertion loss and a 24 dB isolation, which could separate the transmitted signal and the reflected signal. The transmission line (RG316, Kingsignal Inc., Shenzhen, China) had a path loss of 1.65 dB·m^−1^ and a path delay of 4.7 ns·m^−1^. Its main function was delaying the reflected signal to avoid aliasing with the leaked signal from the transmitter to the receiver. During the measuring process, the UWB module generated a UWB signal to be transmitted to the circulator port 1, and the circulator sent the signal from the port 2 (isolated port 3) through the transmission line to the moisture sensor. After that, the signal was reflected at the end face of the coaxial waveguide, and the reflected signal transmitted to the circulator port 2 through the transmission line, and the circulator sent the reflected signal from the port 3 to the receiver.

**Figure 7:**
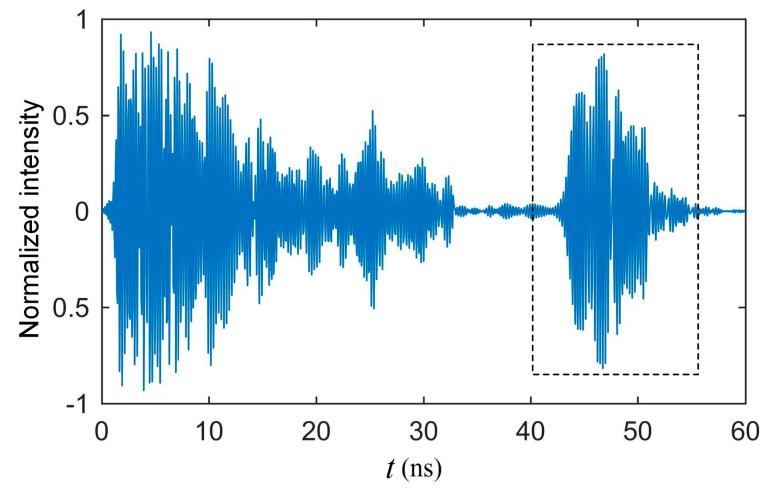
An example of the UWB radar module receiving signal.

Figure 7 shows an example of a received signal when there was no grain in the sampler. The signal between 0 and 35 ns is the leaked signal from the transmitter to the receiver. The signal inside the dashed line box is the reflection signal from the proposed sensor, which is separated from the leaked signal. This represents that the time delay generated by the transmission line successfully avoid aliasing.

Only the signal in the dashed line box was useful to the measurement, so we extracted the signal between 40 ns and 55 ns as the reflected signal *g(t)* in this paper. A virtual origin position $O^{\prime }$ in the transmission line shown in Figure 6b was created, which corresponded to the reflected signal at 40 ns. *G* was the Fast Fourier Transform of *g(t)*. Assuming the incident signal at the position $O^{\prime }$ was *j(t)*, and its Fast Fourier Transform was *J*, the reflection coefficient at the position $O^{\prime }$ can be expressed as follows:

(9) $$\Gamma_{o^{\prime }}^{TEM-I}=\frac{G}{J}$$

If the waveguide between position O and position $O^{\prime }$ was seen as a two-port network, the reflection coefficient at the position $O^{\prime }$ can be calculated from the reflection coefficient at the position O by following equation [33].

(10) $$\Gamma_{o^{\prime }}^{TEM-I}=S_{11}+\frac{S_{12}S_{21}\Gamma_{o}^{TEM-I}}{1-S_{22}\Gamma_{o}^{TEM-I}}$$

where $S_{11}$, $S_{12}$, $S_{21}$ and $S_{22}$ are S-parameters of the two-port network between position O and position $O^{\prime }$. By bringing Equation (9) into Equation (10), we get the following equations:

(11) $$G=\frac{\beta_{1}+\beta_{2}\Gamma_{o}^{TEM-I}}{1-\beta_{3}\Gamma_{o}^{TEM-I}}$$

(12) $$\beta_{1}=JS_{11}$$

(13) $$\beta_{2}=J\left(S_{12}S_{21}-S_{11}S_{22}\right)$$

(14) $$\beta_{3}=S_{22}$$

Except for G and $\Gamma_{o}^{TEM-I}$, $\beta_{1}$, $\beta_{2}$, and $\beta_{3}$ in Equation (11) are not dependent on the measured object. For a certain frequency, they are constants. After the constants are determined, the reflection coefficient of the end of the coaxial waveguide ($\Gamma_{o}^{TEM-I}$) can be calculated from G by using Equation (11).

### 2.5. The Method of Calculating the Moisture Content from the Complex Permittivity

In this paper, the density-independent calibration function (1) was adopted to calculate the moisture content. The calibration function is a general method for calculating the moisture content of grain from the complex coefficient, which can eliminate the influence of the bulk density. In addition, it had no relationship with how the complex permittivity was measured. In general, if the complex permittivity of grain can be measured by any method, the calibration function can be used to calculate the grain moisture content from the complex permittivity. We formed Equation (15) by bring Equation (1) into Equation (2). It can be used for calculating the moisture content of grain from its corresponding complex permittivity:

(15) $$W=b_{1}\sqrt{\frac{\varepsilon^{\prime \prime }}{\varepsilon^{\prime }\left(a_{f}\varepsilon^{\prime }-\varepsilon^{\prime \prime }\right)}}+b_{2}T+b_{3}$$

where $a_{f}$ is a frequency factor, which is related to the microwave frequency. It is a constant for a given variety of grain at a given frequency. T is temperature (°C), W is the percentage of moisture (wet basis). $b_{1}$, $b_{2}$ and $b_{3}$ are three undetermined coefficients. As Equation (1), the expression $\sqrt{\varepsilon^{\prime \prime }/\varepsilon^{\prime }\left(a_{f}\varepsilon^{\prime }-\varepsilon^{\prime \prime }\right)}$ can be denoted by ψ, which is called the density-independent calibration function. Although the complex permittivity of grain in the frequency range of 3.1–4.8 GHz was measured, Equation (15) adopted only the complex permittivity of a single frequency to calculate the moisture content. The performance of Equation (15) is related to the frequency and the grain type. The optimal frequency for different grain types is different. To improve the accuracy, the optimal frequency for each grain type should be determined through the calibration process. Since the UWB radar module measures the wideband reflection coefficient, the complex permittivity of grain obtained is a spectrum from 3.1 to 4.8 GHz. The moisture determination method described in this paper thus has a good performance for multiple types of grains.

### 2.6. Summary of the Proposed Method

**Figure 8:**
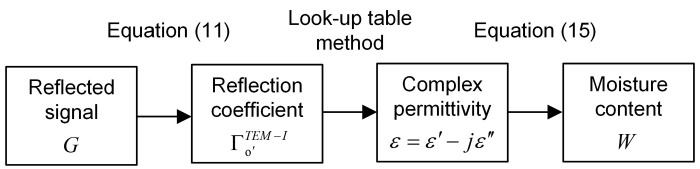
Summary diagram of the proposed moisture content measuring method.

As shown in Figure 8, the proposed moisture content measuring method can be summarized as follows.

(1) Load the sample. 30 mL grain sample can be easily sampled and loaded in the sensor just by inserting the proposed sensor into the grain bulk. Because the circular waveguide designed can be used as the grain sampler and the sample holder.
(2) Collect the reflected signal *g(t)* by using the UWB radar module and calculate *G* by the Fast Fourier Transform.
(3) Calculate the reflection coefficient from the reflected signal in the frequency domain by using Equation (11).
(4) Calculate the complex permittivity of grain from the reflection coefficient by look-up table method proposed in Section 2.3.
(5) Calculate the moisture content from the complex permittivity by using Equation (15).

**Figure 9:**
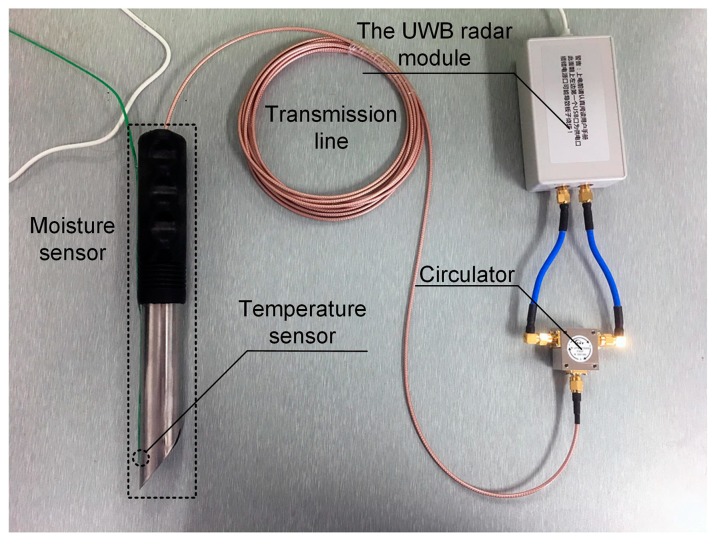
The photograph of the developed device for measuring the moisture content of different types of grains.

Based on the proposed method, a photograph of the developed measuring device is shown in Figure 9.

## 3. Calibration Method

There are two equations that must be calibrated. Therefore, the calibration consists of two steps. The first step is calibrating Equation (11). There are three parameters $\beta_{1}$, $\beta_{2}$, and $\beta_{3}$. Therefore, three standard materials with known complex permittivity can be used to achieve the calibration. In this paper, we firstly fixed the proposed sensor vertically and made the sample holder up. Then filled the sampler with air, methanol at 20 °C, or ethanol at 20 °C. Their corresponding reflected signals in the frequency domain ($G_{1}$, $G_{2}$, and $G_{3}$) were obtained, and their corresponding reflection coefficients of the end of coaxial waveguide ($\Gamma_{1}^{TEM-I}$, $\Gamma_{2}^{TEM-I}$, and $\Gamma_{3}^{TEM-I}$) were calculated by using Equation (8). The complex permittivity of air equals one, and the complex permittivity of methanol and ethanol can be obtained from the tables of dielectric dispersion data for pure liquids [34]. The three sets of data were brought into Equation (11), and the three functions were expressed in matrix form as follows:

(16) $$\left[\begin{array}{c} \beta_{1}\\ \beta_{2}\\ \beta_{3} \end{array}\right]={\left[\begin{array}{ccc} 1 & \Gamma_{1}^{TEM-I} & G_{1}\Gamma_{1}^{TEM-I}\\ 1 & \Gamma_{2}^{TEM-I} & G_{2}\Gamma_{2}^{TEM-I}\\ 1 & \Gamma_{3}^{TEM-I} & G_{3}\Gamma_{3}^{TEM-I} \end{array}\right]}^{-1}\left[\begin{array}{c} G_{1}\\ G_{2}\\ G_{3} \end{array}\right]$$

The three parameters $\beta_{1}$, $\beta_{2}$, and $\beta_{3}$ can be determined by solving Equation (16). This calibration step was only needed to be done one time after the proposed moisture measuring device was manufactured. 

The second step is calibrating Equation (15). There is the optimal frequency ($f_{0}$), the frequency factor $a_{f}$, and three coefficients ($b_{1}$, $b_{2}$, $b_{3}$) that need to be determined. Firstly, we created a calibration data set. For a certain type of grain, four samples with different moisture contents were prepared. Their standard moisture content ($W_{1}$, $W_{2}$, $W_{3}$, $W_{4}$) was determined by oven method (ISO 712-2009), and their complex permittivity spectra at 20 °C ($\varepsilon_{1-20}$, $\varepsilon_{2-20}$, $\varepsilon_{3-20}$, $\varepsilon_{4-20}$) were measured, and their complex permittivity spectra at 30 °C ($\varepsilon_{1-30}$, $\varepsilon_{2-30}$, $\varepsilon_{3-30}$, $\varepsilon_{4-30}$) were measured. w denoted a vector $\left[\begin{array}{cccc} W_{1} & W_{2} & W_{3} & W_{4} \end{array}\right]$, and $ep_{20}$ denoted a vector $\left[\begin{array}{cccc} \varepsilon_{1-20} & \varepsilon_{2-20} & \varepsilon_{3-20} & \varepsilon_{4-20} \end{array}\right]$, and $ep_{30}$ denoted a column vector $\left[\begin{array}{cccc} \varepsilon_{1-30} & \varepsilon_{2-30} & \varepsilon_{3-30} & \varepsilon_{4-30} \end{array}\right]$. The linear relationship between ψ and w can be used to determine $a_{f}$ and $f_{0}$. We formed an objective function as follows:

(17) $${\tilde{a}}_{f_{0}}\left(f_{0}\right)=\underset{\begin{array}{c} a_{f}\in [0,2]\\ f\in [3.1\mathrm{GHz},4.8\mathrm{GHZ}] \end{array}}{\arg\max}r(w,\psi (ep_{20}(f),a_{f}))$$

where ${\tilde{a}}_{f_{0}}$ is the optimal value of $a_{f}$ at optimal frequency $f_{0}$. $ep_{20}(f)$ denotes the vector $ep_{20}$ at the frequency f. $\psi (ep_{20}(f),a_{f})$ denotes the vector of ψ, which obtained by bringing $ep_{20}(f)$ and $a_{f}$ into Equation (1). $r(w,\psi (ep_{20}(f),a_{f}))$ represents the linear correlation coefficient of w and $\psi (ep_{20}(f),a_{f})$ of calibration data set. The objective function aims to find the best combination of $a_{f}$ and f to make the linear correlation coefficient take the maximum value. In this paper, the range of $a_{f}$ was discretized from zero to one in steps of 0.0001, and the range of f was discretized from 3.1 to 4.8 GHz in the steps of 0.1 GHz. The calibration data set w and $ep_{20}$ were brought into the objective function (17), and tried every combination of $a_{f}$ and f to find the optimal values (${\tilde{a}}_{f_{0}}$ and $f_{0}$) which made the linear correlation coefficient take the maximum. Finally, other parameters ($b_{1}$, $b_{2}$, $b_{3}$) can be obtained by bringing every calibration data into Equation (15) and a matrix function can be formed as follows:

(18) $$Ab=w^{\prime }$$

(19) $$A=\left[\begin{array}{ccc} \psi_{1-20} & T_{20} & 1\\ \psi_{2-20} & T_{20} & 1\\ \psi_{3-20} & T_{20} & 1\\ \psi_{4-20} & T_{20} & 1\\ \psi_{1-30} & T_{30} & 1\\ \psi_{2-30} & T_{30} & 1\\ \psi_{3-30} & T_{30} & 1\\ \psi_{4-30} & T_{30} & 1 \end{array}\right]$$

(20) $$b={\left[\begin{array}{ccc} b_{1} & b_{2} & b_{3} \end{array}\right]}^{T}$$

(21) $$w^{\prime }={\left[\begin{array}{cccc} \begin{array}{cccc} W_{1} & W_{2} & W_{3} & W_{4} \end{array} & W_{1} & W_{2} & \begin{array}{cc} W_{3} & W_{4} \end{array} \end{array}\right]}^{T}$$

where $T_{20}$ equals 20, and $T_{30}$ equals 30. $\psi_{1-20}$, $\psi_{2-20}$, $\psi_{3-20}$, $\psi_{4-20}$, $\psi_{1-30}$, $\psi_{3-30}$ and $\psi_{4-30}$ denote the value ψ by bringing $\varepsilon_{1-20}$, $\varepsilon_{2-20}$, $\varepsilon_{3-20}$, $\varepsilon_{4-20}$, $\varepsilon_{1-30}$, $\varepsilon_{2-30}$, $\varepsilon_{3-30}$, and $\varepsilon_{4-30}$ into Equation (1), respectively. The matrix function can be solved by the least squares method as follows:

(22) $$b={\left(A^{T}A\right)}^{-1}A^{T}w$$

For each type of grain, this calibration step was needed to be done one by one.

## 4. Sample Preparation and Measurement

For this study, about 2 kg of hard white winter wheat (cultivar: Hemai-20) with initial moisture of 11.0% (wet basis) were obtained from Keyuan Seed Industry Co., Ltd. (Shandong, China). About 2 kg of winter barley (cultivar: Xiyin-2) with initial moisture of 10.2% (wet basis) were obtained from Ruifeng Seed Industry Co., Ltd. (Gansu, China). About 2 kg of rough rice (cultivar: Liangyou-3218) with initial moisture of 11.0% (wet basis) were obtained from Kelongyu Seed Industry Co., Ltd., (Hunan, China). Each type of grain was divided into 20 samples of 100 g each and these were conditioned to 20 different moisture contents using the method described by Trabelsi [19] and Nelson [35]. The samples with moisture contents above their initial values were prepared by spraying grains with distilled water and storing in plastic bags for 72 h at 4 °C to equilibrate. The samples with moisture contents below their initial values were prepared by drying at 80 °C in a convection oven for 2 h to 72 h. All samples were manually mixed regularly to ensure even distribution of moisture and were equilibrated to room temperature (25 °C) overnight before measurements. The moisture contents were determined by grinding and drying in triplicate, 5 g samples in a convection oven at 130 °C for 90 min (ISO 712-2009) [4]. The standard deviations of each moisture were less than 0.17. The moisture contents determined by the oven drying method were considered true values. 

**Figure 10:**
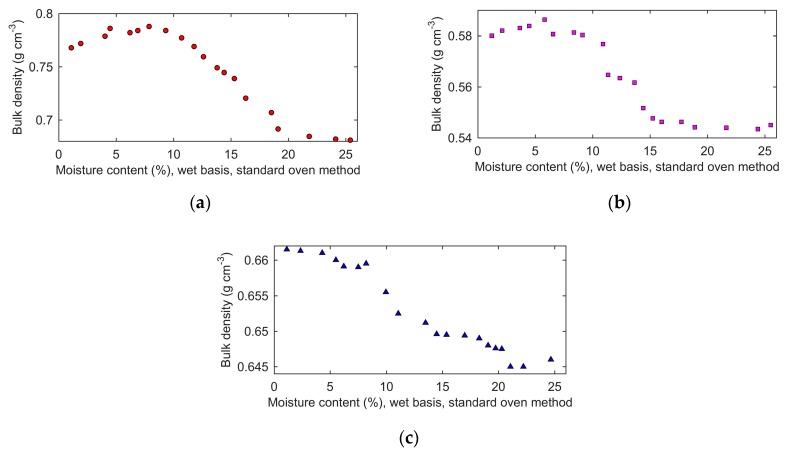
Bulk density of the prepared grain samples dependent of moisture content: (**a**), wheat; (**b**), rough rice; (**c**), barley.

Bulk densities of all prepared samples were measured, and the results are shown in Figure 10. When the moisture content was less than 8%, wet basis, the bulk density of wheat and rough rice increased slightly with the increase of moisture. When the moisture content was more than 8%, wet basis, the bulk density of wheat and rough rice decreased sharply at first and then decreased slightly. 

The bulk density of barley decreased with increasing moisture content. At the same moisture content, the bulk density of wheat was larger than that of rough rice, and the bulk density of rough rice was larger than that of barley. 

In addition, the length of wheat kernel, rough rice kernel, and barley kernel were measured. The average length increased with the increase of moisture content. The length of wheat was from 6.1 mm to 6.7 mm, and the length rough rice was from 7.4 mm to 8.2 mm, the length of barley was from 8.7 mm to 9.6 mm.

**Figure 11:**
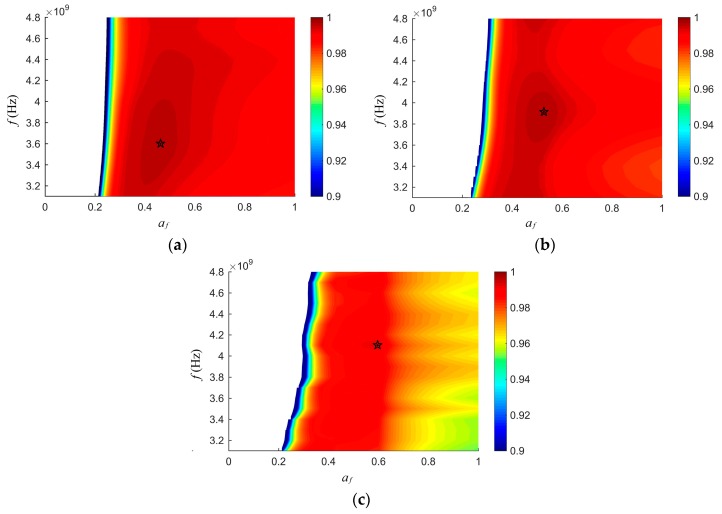
The frequency factor $a_{f}$ and f dependence of the value of the objective function (17): (**a**), wheat; (**b**), rough rice; (**c**), barley.

Based on the proposed calibration method, four samples with moisture contents approximately 5%, 10%, 15%, and 20% were selected from the prepared samples as calibration samples for each type of grain (wheat, rough rice, and barley). Figure 11a,b,c illustrate the frequency factor $a_{f}$ and f dependence of the value of the objective function (17) for wheat, rough rice, and barley, respectively. 

The blank region on the left side of the figure was the forbidden region. When $a_{f}$ was too small, the expression $\varepsilon^{\prime \prime }/\varepsilon^{\prime }\left(a_{f}\varepsilon^{\prime }-\varepsilon^{\prime \prime }\right)$ was smaller than zero, which made ψ an imaginary number. It was meaningless that ψ was an imaginary number, so the blank region on the left side of the figures indicated the forbidden region. The pentagram in each figure denotes the optimal combination of $a_{f}$ and f. It can be seen there is the best combination for each type of grain, and the optimal values are shown in Table 2.

After calibration, 20 wheat samples, 20 barley samples, and 20 rough rice samples were sequentially measured by using the developed moisture measuring device. The measurements were carried out at the room temperature (25 °C). The grain samples were placed in many plastic cups, and the sensor was inserted vertically into the grain, and each sample was measured five times.

## 5. Results and Discussion

### 5.1. Measured Moisture Contents Results

**Figure 12:**
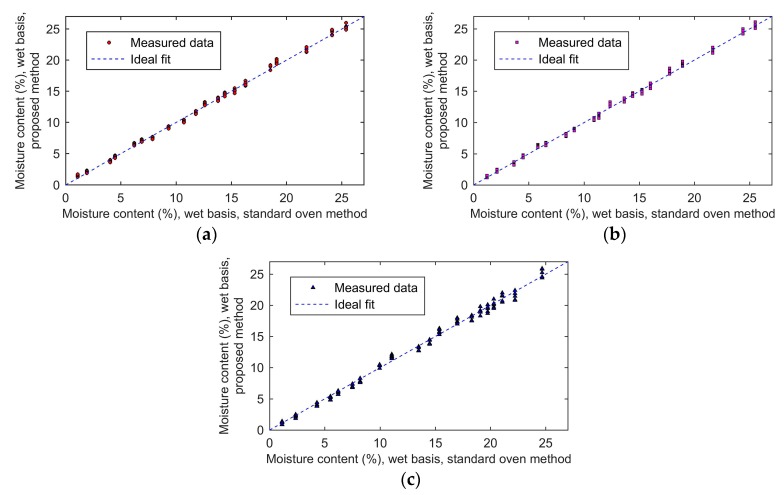
The moisture content of measured results: (**a**), wheat; (**b**), rough rice; (**c**), barley.

The moisture content results measured with the developed device and moisture contents determined by oven drying method for wheat, rough rice, and barley are compared in Figure 12a,b,c, respectively. 

Firstly, it can be seen that the measured results of the proposed method agree well with the values of the standard moisture content from the oven drying method. The R^2^ (coefficients of determination [36]) was used to illustrate the level of agreement between the measured data and the standard data. The range of R^2^ is from zero to one. The larger the value of R^2^, the better the measured data agree with the standard data. When the value of R^2^ equals one, the measured data are equal to the standard data. The R^2^ of wheat was 0.997, the R^2^ of rough rice was 0.994, and the R^2^ of barley was 0.993. Second, the maximum absolute error of the wheat was 1.1%, the maximum absolute error of barley was 1.4%, and the maximum absolute error of rough rice was 1.0%. The mean relative errors of wheat, rough rice, and barley were 4.8%, 4.1%, and 4.8%, respectively. After averaging five results of each sample, the maximum absolute error of the wheat reduced to 0.6%, of the rough rice reduced to 0.5%, and of the barley reduced to 0.7%. As a result, multiple measurements can improve the accuracy of the proposed method. Finally, the standard deviation of each sample increased with the increase in moisture content. The maximum standard deviation of wheat was 0.407, which occurred when the moisture was 24.1%. The maximum standard deviation of barley was 0.680, which appeared when the moisture was 24.7%. The maximum standard deviation of rough rice was 0.423, which occurred when the moisture was 21.7%.

Since the effectiveness of measuring moisture content in some published literature [13,37] was evaluated by calculating the SEP (standard error of performance) defined as follows:

(23) $$\mathrm{SEP}=\sqrt{\frac{1}{n-1}\sum_{i=1}^{n}{\left(W_{i}^{standard}-W_{i}^{measured}\right)}^{2}}$$

where *n* is the number of samples, $W_{i}^{measured}$ denotes the measured moisture content of the *i*-th sample, and $W_{i}^{standard}$ denotes the standard moisture content of the *i*-th sample. The accuracy of the moisture content of wheat, rough rice, and barley measured by the proposed method versus methods of literature are shown in Table 3, Table 4 and Table 5, respectively. 

The proposed method achieved better accuracy of measuring the moisture of wheat and rough rice than the cited literature. The accuracy of measuring the moisture of barley was similar to the literature [37]. Both published articles [16] and [38] used the free space method to measure the complex permittivity of grain. The electromagnetic waves propagated in free space. Although narrow beam antennas and larger sample holder were adopted, there was still a part of electromagnetic waves passing through the edges of the sample holder instead of penetrating grain sample. This would introduce errors into the measurement. In addition, larger sample holder made the device difficult to carry and use in the field conditions. The [2] parallel transmission lines were used as a probe. The electromagnetic field of this method was open in space. If the volume of the grain sample was not large enough, the electromagnetic wave would leak out of the grain sample, and the result would be affected by the environment. Reference [37] used a coaxial sample holder to measure the impedance. The electromagnetic field existed in a limited space, which avoided external interference. Therefore, it obtained better accuracy. Compared with the cited literature, the proposed method gave good accuracy for wheat, rough rice, and barley. In addition, the measuring device was small and portable. The high accuracy of the proposed method in this paper was derived from three aspects. First, the proposed sensor was a closed system. The electromagnetic field existed only inside the circular waveguide, which can avoid external interference. Secondly, the electromagnetic field model of the proposed sensor was developed based on the mode matching method. This method was an analytical way to describe the relationship between the complex permittivity and the reflection coefficient. Its accuracy was much higher than the approximate model. In addition, the grain samples used in this paper were all clean seeds, and the impurity content was very low, which avoided the influence of impurities on the results during the measurement.

### 5.2. Verification of the Performance of Measuring the Complex Permittivity

**Figure 13:**
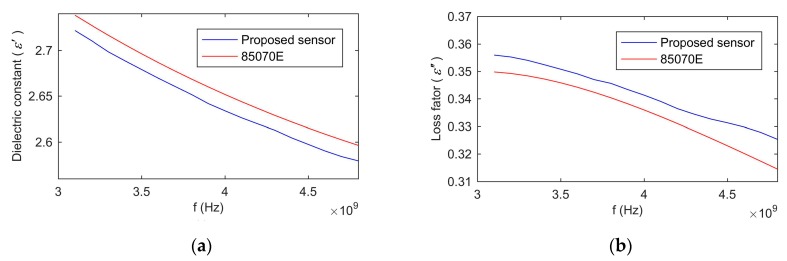
The complex permittivity of decanol measured by the proposed sensor versus probe 85070E at 20 °C: (**a**), dielectric constant; (**b**), loss factor.

Since the complex permittivity is an important intermediate variable of the proposed method in this paper, it is important to verify the accuracy of the measurement of the complex permittivity. Decanol was adopted as the standard material for test because its range of complex permittivity was very close to the grain. An 85070E dielectric probe (Keysight Technologies) was used as a standard method. The method of calculating the complex permittivity by the proposed sensor included three steps. First, the reflected signal G was measured by the ultra-wideband radar module. Then, the reflection coefficient of the sensor was calculated by Equation (11). Finally, the complex permittivity was calculated by the look-up table method. Figure 13 showed the dielectric constant and loss factor of decanol measured by the proposed sensor versus the probe 85070E at 20 °C. The measured results by the proposed sensor were consistent with the results measured by the probe 85070E. The mean relative error of the dielectric constant was 0.6%, and the mean relative error of the loss factor was 1.9%. Therefore, the complex permittivity measured by the proposed sensor had good precision.

### 5.3. Influences of the Moisture Content on Reflection Coefficient, Complex Permittivity, and Reflected Signal

**Figure 14:**
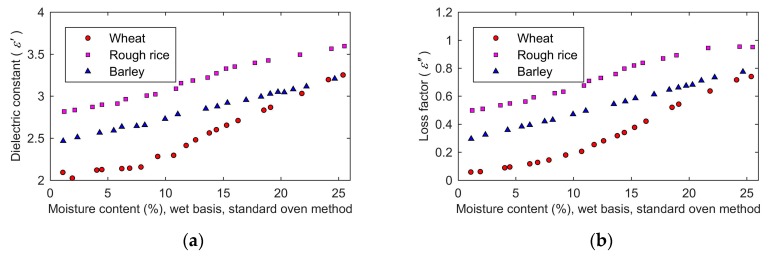
The moisture content dependence of the complex permittivity: (**a**), dielectric constant; (**b**), loss factor.

Due to the complex permittivity, the reflection coefficient and the reflected signal are intermediate variables in the measurement process in this paper, it is important to analyze the influence of moisture content on them. The effects of moisture content on the dielectric constant and loss factor are shown in Figure 14a,b, respectively. Since the optimal frequencies for wheat, rough rice, and barley are 3.6 GHz, 4.0 GHz, and 4.1 GHz, the data of wheat, rough rice, and barley in Figure 14a,b was measured at 3.6 GHz, 4.0 GHz, and 4.1 GHz, respectively. Both the dielectric constant and the loss factor increased with the increase of the moisture content. Since the dielectric constant and loss factor of water were much larger than that of dry grain, the moisture content had a great influence on the complex permittivity of grain. The dielectric constant and loss factor of grain rose with the increase of the moisture content. In addition, the relationship between complex permittivity and the moisture content was not linear. The volume of the grain kernel rose with the increase of moisture content. That led to the porosity change, which affected the volume rate of air in the sample holder. This factor made the influence of moisture content on the complex permittivity of grain as non-linear.

**Figure 15:**
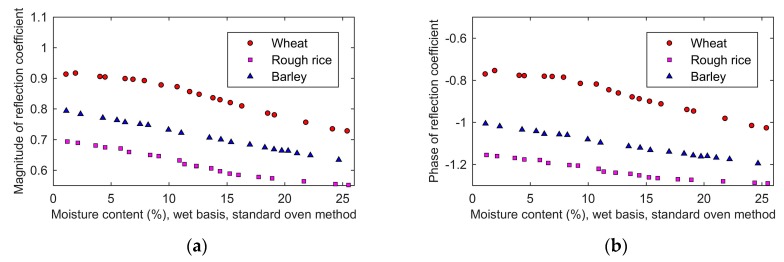
The moisture content dependence of the reflection coefficient: (**a**), magnitude; (**b**), phase.

Figure 15 shows the effect of the moisture content on the reflection coefficient of the proposed sensor. Both the magnitude and phase of the reflection coefficient decreased with the increasing moisture. The influence of moisture content on the reflection coefficient was caused by the change of complex permittivity. The change of complex permittivity made the wave impedance of the grain in the proposed sensor change, which affected the reflection coefficient. The proposed model expressed by Equation (4) in this paper can accurately describe the relationship between the complex permittivity and the reflection coefficient.

**Figure 16:**
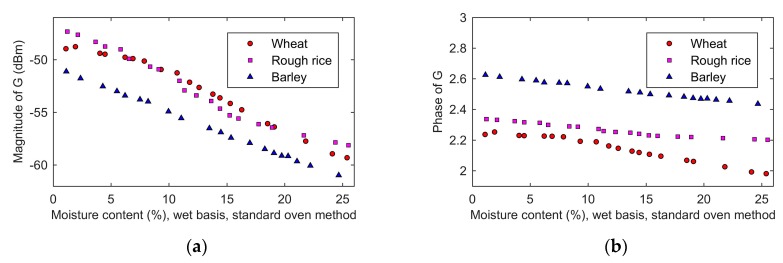
The moisture content dependence of the reflected signal: (**a**) magnitude; (**b**) phase.

Figure 16a,b shows the moisture content dependence of the magnitude and phase of the reflected signal *g*(*t*). The magnitude of the reflected signal was between −46 dBm and −60 dBm, and it decreased with the increase of the moisture content. As the moisture increased, more electromagnetic energy entered the grain, and less electromagnetic energy reflected back. This was consistent with the decrease in the magnitude of the reflection coefficient as the moisture increases (Figure 15a). The phase of the reflected signal decreased as the moisture content increased. Since the incident signal was constant and the phase of the reflection coefficient was equal to the phase of the reflected signal minus the phase of the incident signal, the phase of the reflected signal changed in the same direction with the reflection coefficient. This was consistent with the results that both the phase of the reflected signal and the reflection coefficient decreased as the moisture content increased. The quantitative relationship between the moisture content and the reflected signal was complicated. It can be depicted by combining Equations (15), (4), and (11). Equation (15) represented the relationship between the moisture content and the complex permittivity, and Equation (4) represented the relationship between the complex permittivity and the reflection coefficient, and Equation (11) represented the relationship between the reflection coefficient and the reflected signal. Consequently, calculating the reflected signal from the complex permittivity or calculating the complex permittivity from the reflected signal can be achieved by Equations (15), (4), and (11). 

### 5.4. Verification of the Method of Determining the Frequency Factor by Optimizing the Objective Function

**Figure 17:**
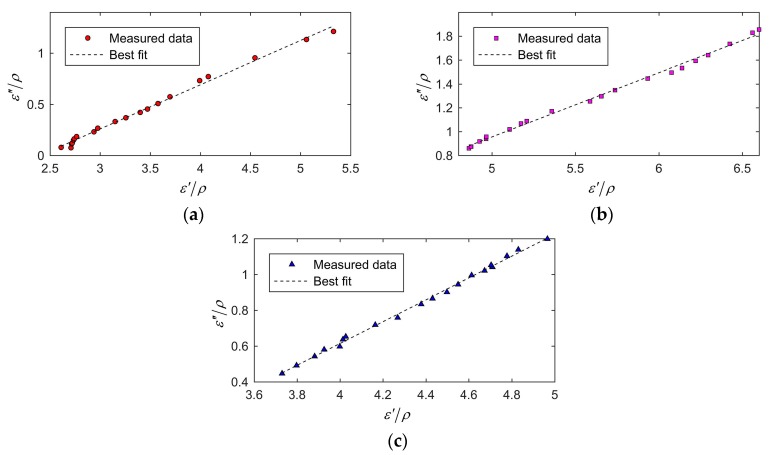
Linear regression analysis on the data: (**a**), wheat at 3.6 GHz; (**b**), rough rice at 4.0 GHz; (**c**), barley at 4.1 GHz.

The determination of the frequency factor $a_{f}$ in literature [16] was determined by analyzing the data of $\varepsilon^{\prime }/\rho$ and $\varepsilon^{\prime \prime }/\rho$ by linear regression, and the value of $a_{f}$ equaled the slope of the linear regression function. However, this method required measuring bulk density, which needed additional equipment. In this paper, an objective function (17) was proposed to determine $a_{f}$ by optimization based on the linear relationship between density-independent calibration function ψ and moisture content W. It had the advantage of avoiding the measurement of bulk density during calibration and improving efficiency. To verify the validity of the method used in this paper, we performed a linear regression analysis on data $\varepsilon^{\prime }/\rho$ and $\varepsilon^{\prime \prime }/\rho$, and the results are illustrated in Figure 17a,b,c, for wheat at 3.6 GHz, rough rice at 4.0 GHz, and barley at 4.1 GHz, respectively. The slopes of the linear regression function were 0.4625, 0.5388, and 0.6094 for wheat, rough rice, and barley, respectively. The relative errors between $a_{f}$ determined by optimization and determined by the method in the literature [16] were 0.7%, 0.8%, and 1.8% for wheat, rough rice, and barley, respectively. $a_{f}$ obtained by the method we used were consistent with $a_{f}$ obtained by the methods in the literature. Therefore, the parameter estimation method can accurately determine $a_{f}$ without measuring bulk density.

### 5.5. Influence of the Isolation Layer on the Stability

**Figure 18:**
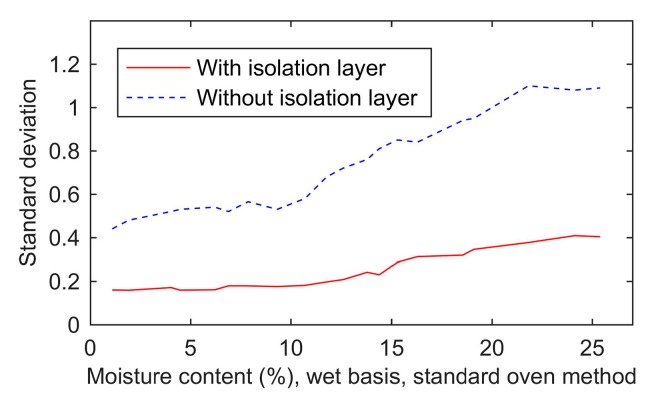
The influence of the isolation layer on the standard deviation.

To verify the effect of the isolation layer on the stability, 20 wheat samples’ moisture contents were measured by a moisture sensor without an isolation layer. Each sample was measured five times, and the standard deviation of each sample was computed. The results are shown in Figure 18. The standard deviations of the measured results using the sensor with the isolation layer were significantly decreased by 0.52 on average comparing to the standard deviations without the isolation layer. Therefore, the isolation layer improved the stability.

In this paper, the moisture content was calculated from the complex permittivity, and the complex permittivity was calculated from the reflection coefficient, and the reflection coefficient was measured by the UWB radar module. The complex permittivity of grain is the complex permittivity of the mixture of grain kernels and air. Consequently, the porosity has a large effect on the complex permittivity of the mixture. It means that the complex permittivity of the mixture is influenced by the volume rate of grain kernels and air. Unfortunately, the distribution of porosity in grain is not uniform. For example, there is 1 L wheat, and let us assume its standard porosity equals 50%. If 1 mL wheat is sampled to measure its porosity, the value will be random. It may be greater than 50% or less than 50%, which means that the variance of the measured result is large. If 100 mL wheat sample is sampled to measure the porosity, the result will also be random, but the variance must be smaller than the result of measured using 1 mL sample. If all wheat (1 L) are taken to measure the porosity, the value will be equal to 50%, and the variance will be approximately zero. Therefore, the variance of the porosity decreases with the increasing of the sampling volume. Considering the grain in the sample holder of the proposed sensor to be stratified, the complex permittivity of the first layer, which directly contacts with the inner and outer conductors of the coaxial waveguide, has a greater influence on the reflection coefficient than the other layers. Since the volume of this layer is very small, the randomness of porosity is strong. This indicates that the complex permittivity of this layer has strong randomness. However, the layer with strong randomness has a greater influence on the reflection coefficient than other layers. This leads to a large variance in the measured result. The isolation layer can prevent the grain kernels from direct contact with the coaxial inner and outer conductors. As a result, there is no direct circuit from the inner conductor to the outer conductor through the grain kernels. The influence of the porosity’s randomness of the first layer on the measurement can be reduced by the isolation layer.

**Figure 19:**
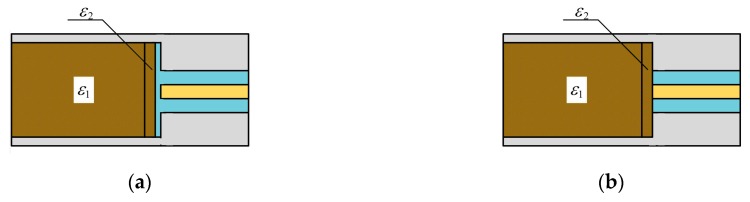
The simulation model for verification effect of the isolation layer: (**a**), with the isolation layer; (**b**), without the isolation layer.

In order to verify the effect of the first layer’s complex permittivity on the reflection coefficient of the proposed sensor, the CST (Dassault Systemes) simulation software was adopted, and the simulation models are shown in Figure 19. Figure 19a shows the sensor with the isolation layer, and the area within 3 mm from the isolation layer was the first layer. The complex permittivity of the first layer was $\varepsilon_{2}$, and the complex permittivity of the rest of the grain was $\varepsilon_{1}$. Figure 19b shows the sensor without the isolation layer, and the area within 3 mm from the opening of the coaxial waveguide is the first layer. Its complex permittivity was $\varepsilon_{2}$, and the complex permittivity of the rest of grain was $\varepsilon_{1}$. 

**Figure 20:**
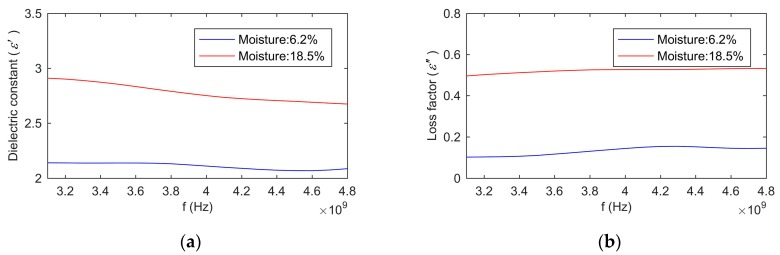
The dielectric constant of wheat with the moisture content of 6.2% and 18.5%: (**a**), dielectric constant; (**b**), loss factor.

We defined three statuses. The status 1 denoted that the complex permittivity of the first layer equaled the complex permittivity of the rest of grain. The status 2 denoted that the complex permittivity of the first layer was 10% smaller than the complex permittivity of the rest of grain. The status 3 denoted that the complex permittivity of the first layer was 10% larger than the complex permittivity of the rest of grain. The prepared wheat with 6.2% and 18.5% moisture content were adopted as the measuring grain for the simulation. Their dielectric constants and loss factors are shown in Figure 20a,b, respectively. The values were measured by the proposed method.

**Figure 21:**
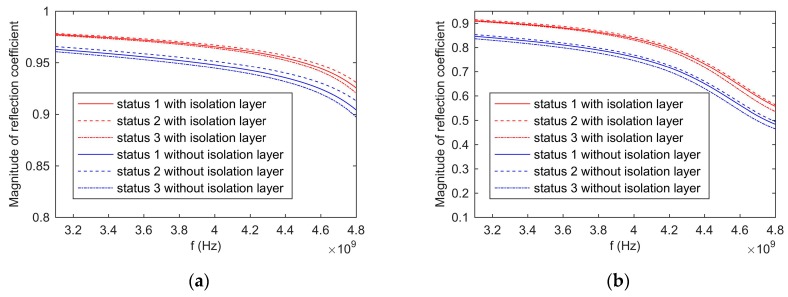
The magnitude of reflection coefficient: (**a**), the moisture content of wheat equals 6.2%; (**b**), the moisture content of wheat equals 18.5%.

Figure 21a shows the simulation results for the moisture content of 6.2%. It was obvious that the changes of the complex permittivity of the first layer had a significant effect on the reflection coefficient of the sensor without the isolation layer. The influence on the reflection coefficient of the sensor with the isolation layer was small. Taking 3.6 GHz as an example, when the complex permittivity of the first layer increased by 10%, the reflection coefficient of the sensor without the isolation layer reduced by 0.29%, and the reflection coefficient of the sensor with the isolation layer reduced by 0.11%. When the complex permittivity of the first layer reduced by 10%, the reflection coefficient of the sensor without the isolation layer increased by 0.31%, and the reflection coefficient of the sensor with the isolation layer increased by 0.13%. 

Figure 21b shows the simulation results for the moisture content of 18.5%. Similar to Figure 21a, the isolation layer reduced the effect of the changes of the complex permittivity of the first layer on the reflection coefficient. Taking 3.6 GHz as an example, when the complex permittivity of the first layer increased by 10%, the reflection coefficient of the sensor without the isolation layer reduced by 1.43%, and the reflection coefficient of the sensor with the isolation layer reduced by 0.39%. When the complex permittivity of the first layer reduced by 10%, the reflection coefficient of the sensor without the isolation layer increased by 0.97%, and the reflection coefficient of the sensor with the isolation layer increased by 0.64%. Comparing Figure 21a with Figure 21b, it can be seen that the changes of the reflection coefficient of the high moisture grain were greater than that of the low moisture grain. 

The results of the simulation agreed with the experimental results shown in Figure 18. The isolation layer can reduce the variance of the measured results. In addition, the variance of the measured results rose with the increase of the moisture content.

**Figure 22:**
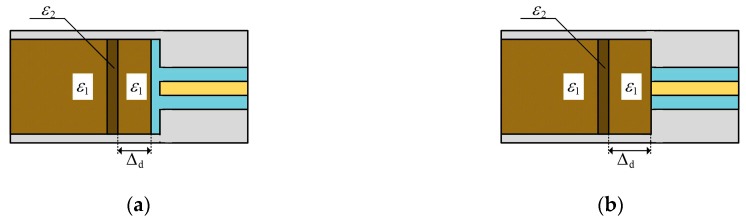
The simulation model for verification the effect of complex permittivity changes in different regions on the reflection coefficient: (**a**), with the isolation layer; (**b**), without the isolation layer.

To further verify the effect of complex dielectric constant changes in different regions on the reflection coefficient, we established the simulation models as shown in Figure 22. The complex permittivity of a 3 mm thick grain layer in the sample holder was $\varepsilon_{2}$, and the complex permittivity of grain in other regions was $\varepsilon_{1}$. For the sensor with the isolation layer, $\Delta_{d}$ denoted the distance between the grain layer and the isolation layer. For the sensor without the isolation layer, $\Delta_{d}$ denoted the distance between the grain layer and the end face of the coaxial waveguide. $\varepsilon_{1}$ equaled the complex permittivity of prepared wheat with the 18.5% moisture content (Figure 20a,b). $\varepsilon_{2}$ was 10% larger than $\varepsilon_{1}$. The simulation evaluated the effect of a 10% increase in the complex permittivity of a 3 mm thick grain layer on the reflection coefficient at 3.6 GHz.

**Figure 23:**
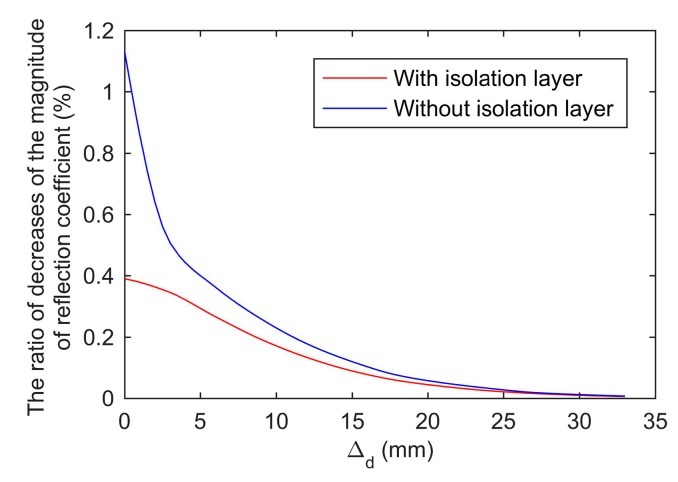
The ratio of decreases of the magnitude of reflection coefficient with the complex permittivity increasing by 10%.

The simulation results were shown in Figure 23. First, with or without the isolation layer, the decreases’ ratio of the magnitude of the reflection coefficient shrank with the increase of the distance $\Delta_{d}$. This was mainly caused by the intensity of the electromagnetic field decreasing with the increase of the distance $\Delta_{d}$. The farther the distance, the weaker the effect of complex permittivity changes on the reflection coefficient. Secondly, the changes of the complex permittivity in the region, which was within 3 mm from the end face of the coaxial waveguide of the sensor without the isolation layer, had a greater influence on the reflection coefficient than other regions. In contrast, the reflection coefficient of the sensor with the isolation layer was less sensitive to the changes of the complex permittivity than the sensor without the isolation layer. It was caused by the isolation layer blocking a part of the electromagnetic wave’s energy. Fortunately, the isolation layer weakened the effect of the changes of the complex permittivity of the first layer on the reflection coefficient. The isolation layer reduced the level of inhomogeneity of the complex permittivity in different regions influencing on the reflection coefficient. As a result, the reflection coefficient measured by the proposed sensor was more accurately related to the complex permittivity of the whole grain in the sample holder rather than the grain near the end face of the coaxial waveguide. In summary, the isolation layer can reduce the variance of the measured results and improve the performance.

### 5.6. Influence of the Pressure on The Measurement

**Figure 24:**
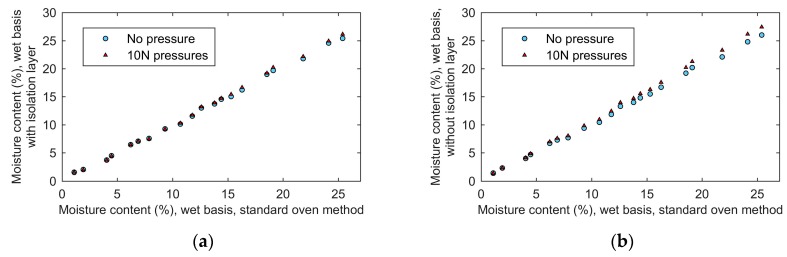
The influence of pressures on the measurement: (**a**), with the isolation layer; (**b**), without the isolation layer.

Since the pressure may be applied to the sensor by hand in practical applications, or due to grain above it, it is important to study the influence of external pressure on the measurement. In this paper, the proposed moisture sensor was firstly inserted vertically into the wheat sample and the moisture was measured without pressure. Then, a 1 kg weight (about 10 N pressure) was placed on the sensor and the moisture was measured. The results are shown in Figure 24a. The measured moisture content increased slightly with the increase in pressure and this increase was higher at higher moisture contents. For wheat sample with 25.4% moisture, the measured result with 10 N pressure was larger than that without pressure by 0.7. For moisture below 5.0%, the 10 N pressure did not noticeably affect the measurement. This is mainly because the pressure compressed the grain in the sampler, which made the proportion of grain kernels in the sampler rise and the volume ratio of grain’s moisture in the sampler became larger. In addition, grain kernels became softer as moisture increased. The higher the moisture content, the easier these can be compressed, and the more significant is the effect of pressure. Therefore, the pressure had a more obvious influence on the measurement of high moisture grain.

In another aspect, the proposed method was not sensitive to the change of bulk density. Bulk density of grain in the sample holder can be obtained by dividing the mass of grain by the volume of the sample holder. The volume of the sample holder was constant. With 10 N pressures on the sensor, more grain kernels were brought into the sample holder. Consequently, the mass of grain increased, and the bulk density of grain in the sample holder increased as well. With the increase of bulk density, few changes can be observed in Figure 24a. It indicated that the proposed method eliminated the influence of bulk density.

In addition, we also studied the influence of pressure on the measurements using the sensor without the isolation layer. The results are shown in Figure 24b. Compared with Figure 24a, it is obvious that the pressure had a more significant influence on the measurements without the isolation layer. Measuring the wheat sample with 25.4% moisture by using the sensor without isolation layer, the 10 N pressure made the measuring result increase by 1.4, which was two times larger than that with the isolation layer. When 10 N pressures were applied to the sensor without the isolation, the pressures were transmitted to the grain through the end face of the coaxial waveguide. Grain kernels in the region, which was near the end face of the coaxial waveguide, were compressed to a great degree. As a result, the volume rate of the moisture in the region increased larger than other regions. In addition, without the isolation layer, the first layer of grain, which was directly contacted with the inner and outer conductors of the coaxial waveguide, had a greater influence on the measured result. Eventually, the measured results of the sensor without the isolation layer were more sensitive to pressures. In contrast, the proposed sensor with the isolation layer can reduce the sensitivity of the measurement to the first layer of grain. Consequently, the influence of the pressure on the measurement was reduced and the performance of the proposed method was improved.

### 5.7. Influence of the Frequency on the Accuracy of the Measured Results

**Figure 25:**
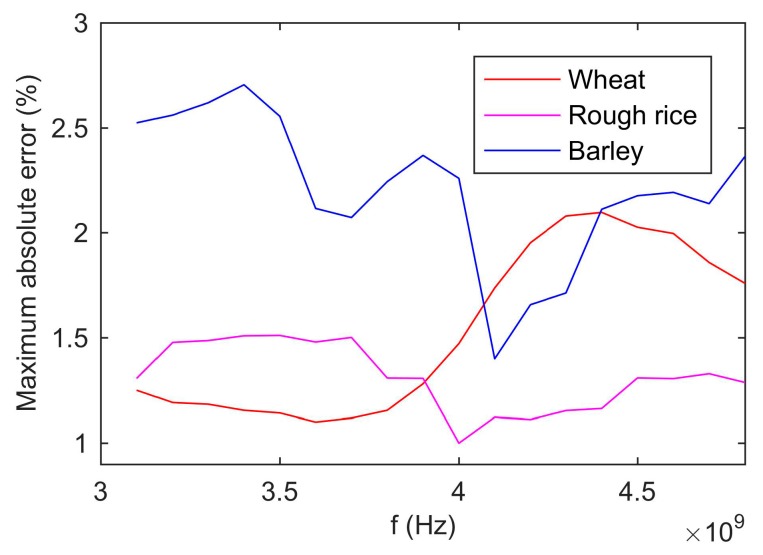
The influence of frequency on the accuracy of the moisture content measured results.

In the paper, the complex permittivity of grain in the frequency range of 3.1–4.8 GHz was measured. However, only the complex permittivity of a single frequency was adopted by Equation (15) to calculate the moisture content. Since the performance of Equation (15) was varied with frequency, selecting the complex permittivity of the optimal frequency could improve the accuracy. Beside, Different types of grain had the different optimal frequency. Consequently, it was necessary to determine the optimal frequency for each grain type. In the range of 3.1 to 4.8 GHz, the frequency was divided into 18 frequency points in steps of 0.1 GHz. The moisture contents were calculated from the complex permittivity at each frequency point. The maximum absolute errors were counted and the results were shown in Figure 25. When the maximum absolute error reached the minimum, its corresponding frequency was the optimal one. The optimal frequency for wheat, rough rice, and barley was 3.6 GHz, 4.0GHz, and 4.1GHz, respectively. This was the same as calibrating results (Table 2 and Figure 11). Since the UWB radar module measured the wideband reflection coefficient, the complex permittivity of grain obtained was a spectrum from 3.1 to 4.8 GHz. It had two advantages. Firstly, the complex permittivity of the optimal frequency could be chosen to improve the accuracy. Secondly, the method had a good performance for multiple types of grains. Since the grain of different type had the different optimal frequency, measuring complex permittivity of wideband had more opportunity to choose the optimal frequency.

## 6. Conclusions

In this paper, a novel portable device was designed, fabricated and evaluated to measure grain moisture content fast and accurately, and the following conclusions can be drawn:

(1) A novel moisture sensor can be designed by combining the grain sampler and the coaxial waveguide. The inner diameter of the sampler should be designed to make all electromagnetic wave modes attenuate. This allows the measurement procedure to require only a small amount of grain sample, and the interferences of the environment can be avoided.
(2) The mode matching method can be used to accurately model the moisture sensor proposed in this paper, and the complex permittivity of grain can be obtained by using the look-up table method.
(3) With UWB radar module, a portable broadband reflection coefficient measuring device can be achieved.
(4) Compared with the accuracy of moisture measurement literature, the proposed method had better precision in measuring wheat and rough rice. The accuracy of measuring barley moisture was similar to the literature. In addition, the proposed method had the advantages of anti-environment interference and portability compared with the methods in the literature.
(5) The pressure on the sensor influenced the measured result, especially for grain with high moisture. Designing an isolation layer between the grain and the coaxial waveguide remarkably reduced the influence and improved the accuracy.

## Figures and Tables

**Table 1:** Dimensions of the sensor.

Symbol	Value (mm)
$R_{a}$	3.0
$R_{b}$	10.0
$R_{}$	15.0
$R_{s}$	16.0
$l_{1}$	30.0
$l_{2}$	1.0
$l_{3}$	40.0

**Table 2:** Parameters of the function for calculating the moisture.

Parameters	Wheat	Rough Rice	Barley
$b_{1}$	44.2478	48.7805	31.5457
$b_{2}$	−0.1018	−0.0976	−0.1136
$b_{3}$	0.1814	0.0829	0.2240
$f_{0}$	3.6 GHz	4.0 GHz	4.1 GHz
${\tilde{a}}_{f_{0}}$	0.4592	0.5347	0.5987

**Table 3:** The accuracy of the moisture content of wheat measured by the proposed method versus methods of literature.

Methods	R^2^	SEP (%)
Proposed method in this paper	0.997	0.14
Method in literature [2] ^1^	0.995	-
Method in literature [12] ^2^	-	0.22
Method in literature [37] ^3^	0.991	-

^1^ Using radio frequency reflection method to measure moisture content of wheat harvested at Raoyang town in Heibei province at 51 to 300 MHz. ^2^ Using microwave free space method to measure moisture content of hard red winter wheat (cultivar: Karl) at 12.3 GHz. ^3^ Using radio frequency impedance to measure moisture content of wheat (cultivar: Olgroo) harvested at the Seoul National University farm at 1 to 10 MHz.

**Table 4:** The accuracy of the moisture content of rough rice measured by the proposed method versus methods of literature.

Methods	R^2^	SEP (%)
Proposed method in this paper	0.994	0.15
Method in literature [37] ^1^	0.985	-
Method in literature [38] ^2^	0.986	0.52

^1^ Using radio frequency impedance to measure moisture content of rough rice (cultivar: Olgroo) harvested at the Seoul National University farm at 1 to 10 MHz. ^2^ Using microwave attenuation and bulk density to measure moisture content of rough rice (cultivar: Hwasung) harvested at the Seoul National University farm at 10.5 GHz.

**Table 5:** The accuracy of the moisture content of barley measured by the proposed method versus methods of literature.

Methods	R^2^	SEP (%)
Proposed method in this paper	0.993	0.30
Method in literature [37] ^1^	0.996	-

^1^ Using radio frequency impedance to measure moisture content of barley (cultivar: Jinyang) harvested at the Seoul National University farm at 1 to 10 MHz.

## Appendix A

In Figure 2b, the moisture sensor can be divided into three regions. Region I denotes the coaxial waveguide, region II denotes isolation layer, and region III denotes sampler. Since only TEM mode can transmit through the coaxial cable, only the reflected electromagnetic field has high-order modes (TM mode and the TE mode). According to the study [39], the electromagnetic fields of high-order modes should be axisymmetric fields, for the coaxial waveguide is an axisymmetric structure. Therefore, the electromagnetic fields of the high-order modes in this paper only need to consider the TM_0n_ and the TE_0n_ modes. The transverse electric and magnetic fields in the region I can be expanded by modes as follows:

(A1) $$\begin{array}{l} {\vec{E}}_{I}=\vec{r}\left[\exp\left(-\gamma^{TEM-I}z\right)+\Gamma^{TEM-I}\exp\left(\gamma^{TEM-I}z\right)\right]e_{0}^{TEM-I}\\ +\vec{r}\sum_{i=1}^{N}m_{i}\exp\left(\gamma_{i}^{TM-I}z\right)e_{i}^{TM-I}+\vec{\varphi }\sum_{i=1}^{N}n_{i}\exp\left(\gamma_{i}^{TE-I}z\right)e_{i}^{TE-I} \end{array}$$

(A2) $$\begin{array}{l} {\vec{H}}_{I}=\vec{\varphi }Y^{TEM-I}\left[\exp\left(-\gamma^{TEM-I}z\right)+\Gamma^{TEM-I}\exp\left(\gamma^{TEM-I}z\right)\right]e_{0}^{TEM-I}\\ -\vec{\varphi }\sum_{i=1}^{N}m_{i}Y_{i}^{TM-I}\exp\left(\gamma_{i}^{TM-I}z\right)e_{i}^{TM-I}-\vec{r}\sum_{i=1}^{N}n_{i}Y_{i}^{TE-I}\exp\left(\gamma_{i}^{TE-I}z\right)e_{i}^{TE-I} \end{array}$$

where $\vec{r}$ and $\vec{\varphi }$ are unit vector, respectively. $\gamma^{TEM-I}$, $\gamma_{i}^{TM-I}$, and $\gamma_{i}^{TE-I}$ are the propagation constants of the TEM mode, the *i*-th TM mode, and the *i*-th TE mode in region I, respectively. $e_{0}^{TEM-I}$, $e_{i}^{TM-I}$, and $e_{i}^{TE-I}$ are the normalized electric field strength of the TEM mode, the *i*-th TM mode, and the *i*-th TE mode in region I, respectively. $\Gamma^{TEM-I}$ is the reflection coefficient of the TEM mode in region I. $m_{i}$, $n_{i}$ are the *i*-th TM mode’s and the *i*-th TE mode’s proportional coefficients of their corresponding normalized electric field strength, respectively. $Y^{TEM-I}$, $Y_{i}^{TM-I}$, and $Y_{i}^{TE-I}$ are admittance of the TEM mode, the *i*-th TM mode and the *i*-th TE mode in region I, respectively. Regarding isolation layer as a circular waveguide, the transverse electric and magnetic fields in the region II can be expanded by modes as follows:

(A3) $$\begin{array}{l} {\vec{E}}_{II}=\vec{r}\sum_{i=1}^{M}c_{i}\left[\exp\left(-\gamma_{i}^{TM-II}z\right)+\Gamma_{i}^{TM-II}\exp\left(\gamma_{i}^{TM-II}z\right)\right]e_{i}^{TM-II}\\ +\vec{\varphi }\sum_{i=1}^{M}d_{i}\left[\exp\left(\gamma_{i}^{TE-II}z\right)+\Gamma_{i}^{TE-II}\exp\left(\gamma_{i}^{TE-II}z\right)\right]e_{i}^{TE-II} \end{array}$$

(A4) $$\begin{array}{l} {\vec{H}}_{t-II}=\vec{\varphi }\sum_{i=1}^{M}c_{i}Y_{i}^{TM-II}\left[\exp\left(-\gamma_{i}^{TM-II}z\right)-\Gamma_{i}^{TM-II}\exp\left(\gamma_{i}^{TM-II}z\right)\right]e_{i}^{TM-II}\\ +\vec{r}\sum_{i=1}^{M}d_{i}Y_{i}^{TE-II}\left[\exp\left(-\gamma_{i}^{TE-II}z\right)-\Gamma_{i}^{TE-II}\exp\left(\gamma_{i}^{TE-II}z\right)\right]e_{i}^{TE-II} \end{array}$$

where $\gamma_{i}^{TM-II}$, $\gamma_{i}^{TE-II}$ are the propagation constants of the *i*-th TM mode and the *i*-th TE mode in region II, respectively. $e_{i}^{TM-II}$, $e_{i}^{TE-II}$ are the normalized electric field strength of the *i*-th TM mode and the *i*-th TE mode in region II, respectively. $\Gamma_{i}^{TM-II}$, $\Gamma_{i}^{TE-II}$ are the reflection coefficients of the *i*-th TM mode and the *i*-th TE mode in region II, respectively. $c_{i}$, $d_{i}$ are the *i*-th TM mode’s and the *i*-th TE mode’s proportional coefficients of their corresponding normalized electric field strength, respectively. $Y_{i}^{TM-II}$, $Y_{i}^{TE-II}$ are admittances of the *i*-th TM mode and the i-th TE mode in region II, respectively. At position O, the boundary conditions can be expressed as follows:

(A5) $${\vec{E}}_{I}\left(z=0^{-}\right)={\vec{E}}_{\mathrm{II}}\left(z=0^{+}\right)$$

(A6) $${\vec{H}}_{I}\left(z=0^{-}\right)={\vec{H}}_{\mathrm{II}}\left(z=0^{+}\right)$$

Bringing Equation (A1), (A2), (A3) and (A4) into Equation (A5) and (A6), we can find that the reflection coefficient of the TEM mode in region I ($\Gamma^{TEM-I}$) is independent of terms of the TE modes. Consequently, ignoring the TE terms, the following can be obtained.

(A7) $$\left[1+\Gamma^{TEM-I}\right]e_{0}^{TEM-I}+\sum_{i=1}^{N}m_{i}e_{i}^{TM-I}=\sum_{i=1}^{M}c_{i}\left(1+\Gamma_{i}^{TM-II}\right)e_{i}^{TM-II}$$

(A8) $$Y^{TEM-I}\left[1-\Gamma^{TEM-I}\right]e_{0}^{TEM-I}-\sum_{i=1}^{N}m_{i}Y_{i}^{TM-I}e_{i}^{TM-I}=\sum_{i=1}^{M}c_{i}\left(1-\Gamma_{i}^{TM-II}\right)Y_{i}^{TM-II}e_{i}^{TM-II}$$

Multiplying $e_{k}^{TM-II}$ at both side of Equation (A7) and integrating in the region of $\left\{R_{a}\leq r\leq R_{b},0\leq \varphi \leq 2\pi \right\}$, $c_{k}$ can be obtained as Equation (A9) by using the orthogonality of different modes.

(A9) $$c_{k}=\frac{1}{1+\Gamma_{i}^{TM-II}}\left(R_{0k}+\sum_{i=1}^{N}m_{i}R_{ik}\right)$$

(A10) $$R_{ik}=\{\begin{array}{c} \int_{0}^{2\pi }\int_{R_{a}}^{R_{b}}e_{0}^{TEM-I}e_{k}^{TM-II}rdrd\varphi ,i=0\\ \int_{0}^{2\pi }\int_{R_{a}}^{R_{b}}e_{i}^{TM-I}e_{k}^{TM-II}rdrd\varphi ,i\neq 0 \end{array}.$$

Bringing Equation (A9) into Equation (A10) we can get Equation (A11):

(A11) $$Y^{TEM-I}\left[1-\Gamma^{TEM-I}\right]e_{0}^{TEM-I}-\sum_{i=1}^{N}m_{i}Y_{i}^{TM-I}e_{i}^{TM-I}=\sum_{i=1}^{M}c_{i}{\bar{Y}}_{i}^{TM-II}\left(R_{0i}+\sum_{j=1}^{N}m_{j}R_{ji}\right)e_{i}^{TM-II}$$

where ${\bar{Y}}_{i}^{TM-II}$ represents the input admittance of the *i*-th TM mode from position O to region II. Since approximately no electromagnetic wave reflection exists at end of the sampler, the input admittance of the region III is approximately equal to the characteristic admittance. Consequently, regarding the region III as the load of the region II, ${\bar{Y}}_{i}^{TM-II}$ can be obtained as follows:

(A12) $${\bar{Y}}_{i}^{TM-II}=Y_{i}^{TM-II}\frac{Y_{i}^{TM-III}+Y_{i}^{TM-II}\tanh\left(\gamma_{i}^{TM-II}l_{2}\right)}{Y_{i}^{TM-II}+Y_{i}^{TM-III}\tanh\left(\gamma_{i}^{TM-II}l_{2}\right)}$$

where $Y_{i}^{TM-II}$ is the characteristic admittance of the *i*-th TM mode in region II, and $Y_{i}^{TM-III}$ is the characteristic admittance of the *i*-th TM mode in region III. Multiplying $e_{k}^{TM-II}$ at both side of Equation (A11) and integrating in the region of $\left\{R_{a}\leq r\leq R_{b},0\leq \varphi \leq 2\pi \right\}$, Equation (A13) can be obtained by using the orthogonality of different modes.

(A13) $$\left(Y^{TEM-I}-{\bar{Y}}_{k}^{TM-II}\right)R_{0k}=\Gamma^{TEM-I}R_{0k}\left(Y^{TEM-I}+{\bar{Y}}_{k}^{TM-II}\right)+\sum_{i=1}^{N}m_{i}R_{ik}\left(Y_{i}^{TM-I}+{\bar{Y}}_{k}^{TM-II}\right)$$

where k∈[1,M]. Finally, Equation (4) in the Methods section can be obtained by rewriting Equation (A13) in matrix form.

## Appendix B

The admittance of each mode in region I can be obtained by using the following equations.

(A14) $$Y^{TEM-I}=\sqrt{\varepsilon_{r}}/120\pi$$

(A15) $$Y_{i}^{TM-I}=j2\pi f\varepsilon_{0}\varepsilon_{r}/\gamma_{i}^{TM-I}$$

The propagation constant $\gamma_{i}^{TM-I}$ of the *i*-th mode in region I can be obtained by using Equation (A3).

(A16) $$\gamma_{i}^{TM-I}=\sqrt{{\left(k_{i}^{TM-I}\right)}^{2}-\varepsilon_{r}{\left(2\pi f\sqrt{\varepsilon_{0}\mu_{0}}\right)}^{2}}$$

where $k_{i}^{TM-I}$ represents the eigenvalue of the *i*-th TM mode in region I, and its value is the *i*-th root of Equation (A17).

(A17) $$J_{0}\left(k_{i}^{TM-I}R_{a}\right)/J_{0}\left(k_{i}^{TM-I}R_{b}\right)=N_{0}\left(k_{i}^{TM-I}R_{a}\right)/N_{0}\left(k_{i}^{TM-I}R_{b}\right)$$

where $J_{0}$ are $N_{0}$ are the Bessel and Neumann functions of order zero. The wave impedance of the *i*-th mode in region I can be obtained by using the following equation:

(A18) $$Z_{i}^{TM-I}=1/Y_{i}^{TM-I}$$

The cut-off frequency of the *i*-th mode in region I can be obtained by using the following equation [33]:

(A19) $$f_{c-i}^{TM-I}=\frac{C_{0}k_{i}^{TM-I}}{2\pi \sqrt{\varepsilon_{r}}}$$

The admittance of each mode in region II can be calculated by using Equation (A20):

(A20) $$Y_{i}^{TM-II}=j2\pi f\varepsilon_{0}\varepsilon_{r}/\gamma_{i}^{TM-II}$$

The propagation constant $\gamma_{i}^{TM-II}$ of the *i*-th mode in region II can be got by using Equation (A21).

(A21) $$\gamma_{i}^{TM-II}=\sqrt{{\left(k_{i}^{TM-II}\right)}^{2}-\varepsilon_{r}{\left(2\pi f\sqrt{\varepsilon_{0}\mu_{0}}\right)}^{2}}$$

The eigenvalue of the *i*-th TM mode in region II ($k_{i}^{TM-II}$) is the *i*-th root of Equation (A22):

(A22) $$J_{0}\left(k_{i}^{TM-II}R\right)=0$$

The wave impedance of the *i*-th mode in region II can be obtained by using the following equation:

(A23) $$Z_{i}^{TM-II}=1/Y_{i}^{TM-II}$$

The cut-off frequency of the *i*-th mode in region II can be obtained by using the following equation [33]:

(A24) $$f_{c-i}^{TM-II}=\frac{C_{0}k_{i}^{TM-II}}{2\pi \sqrt{\varepsilon_{r}}}$$

The admittance of each mode in region III can be calculated by using Equation (A25):

(A25) $$Y_{i}^{TM-III}=j2\pi f\varepsilon_{0}\varepsilon_{MUT}/\gamma_{i}^{TM-III}$$

The propagation constant $\gamma_{i}^{TM-III}$ of the *i*-th mode in region III can be gotten by using Equation (A26).

(A26) $$\gamma_{i}^{TM-III}=\sqrt{{\left(k_{i}^{TM-III}\right)}^{2}-\varepsilon_{MUT}{\left(2\pi f\sqrt{\varepsilon_{0}\mu_{0}}\right)}^{2}}$$

The eigenvalue of the *i*-th TM mode in region III ($k_{i}^{TM-III}$) is equal to $k_{i}^{TM-II}$. The wave impedance of the *i*-th mode in region III can be obtained by using the following equation:

(A27) $$Z_{i}^{TM-III}=1/Y_{i}^{TM-III}$$

The cut-off frequency of the *i*-th mode in region III can be obtained by using the following equation [33]:

(A28) $$f_{c-i}^{TM-III}=\frac{C_{0}k_{i}^{TM-III}}{2\pi \sqrt{\varepsilon_{MUT}}}$$

If *i* equals zero, the coupling coefficient ($R_{ik}$) can be calculated by bringing Equation (A29) and Equation (A30) into Equation (A10). Check this is the correct number

(A29) $$e_{0}^{TEM-I}=-{\left(r\sqrt{2\pi \ln\left(R_{b}/R_{a}\right)}\right)}^{-1}$$

(A30) $$e_{k}^{TM-II}=-J_{0}^{\prime }\left(k_{k}^{TM-I}r\right)/\sqrt{\pi }J_{0}^{\prime }\left(k_{k}^{TM-I}R\right)R$$

If *i* is not equal to zero, Equation (A10) Replace correct number can be transformed into Equation (A31) by using Green’s theorem.

(A31) $$R_{ik}={\left(k_{i}^{TM-II}\right)}^{2}\int_{0}^{2\pi }\int_{a}^{b}\phi_{i}^{TM-I}\phi_{k}^{TM-II}rdrd\varphi$$

where $\phi_{i}^{TM-I}$ and $\phi_{k}^{TM-II}$ are the normalized potential function of the *i*-th TM mode in region I and region II [33].

(A32) $$\phi_{i}^{TM-I}={\left(\pi C_{i}\right)}^{-\frac{1}{2}}\left[N_{0}\left(k_{i}^{TM-I}R_{b}\right)J_{0}\left(k_{i}^{TM-I}r\right)-J_{0}\left(k_{i}^{TM-I}R_{b}\right)N_{0}\left(k_{i}^{TM-I}r\right)\right]$$

(A33) $$\begin{array}{c} C_{i}={\left\{k_{k}^{TM-I}R_{b}\left[J_{0}\left(k_{k}^{TM-I}R_{b}\right)N_{1}\left(k_{k}^{TM-I}R_{b}\right)-N_{0}\left(k_{k}^{TM-I}R_{b}\right)J_{1}\left(k_{k}^{TM-I}R_{b}\right)\right]\right\}}^{2}\\ -{\left\{k_{k}^{TM-I}R_{a}\left[J_{0}\left(k_{k}^{TM-I}R_{b}\right)N_{1}\left(k_{k}^{TM-I}R_{a}\right)-N_{0}\left(k_{k}^{TM-I}R_{b}\right)J_{1}\left(k_{k}^{TM-I}R_{a}\right)\right]\right\}}^{2} \end{array}$$

(A34) $$\phi_{k}^{TM-II}=J_{0}(k_{i}^{TM-II}r)/\sqrt{\pi }J_{0}^{\prime }\left(k_{k}^{TM-I}R\right)k_{k}^{TM-I}R$$

where $J_{1}$, $N_{1}$, and ${J^{\prime }}_{0}$ are the order one Bessel function, order one Neumann function, and the derivative of order zero Bessel function.
